# Design and Multiobjective Dynamic Optimization of Superheaters for Load-Following Operation in Pulverized Coal Power Plants

**DOI:** 10.1021/acs.iecr.3c02130

**Published:** 2023-12-20

**Authors:** Quang Minh Le, Jinliang Ma, Debangsu Bhattacharyya, Stephen E. Zitney, Anthony P. Burgard

**Affiliations:** † Department of Chemical and Biomedical Engineering, West Virginia University, Morgantown, West Virginia 26506, United States; ‡ National Energy Technology Laboratory, Pittsburgh, Pennsylvania 15236, United States; § National Energy Technology Laboratory, Morgantown, West Virginia 26507, United States

## Abstract

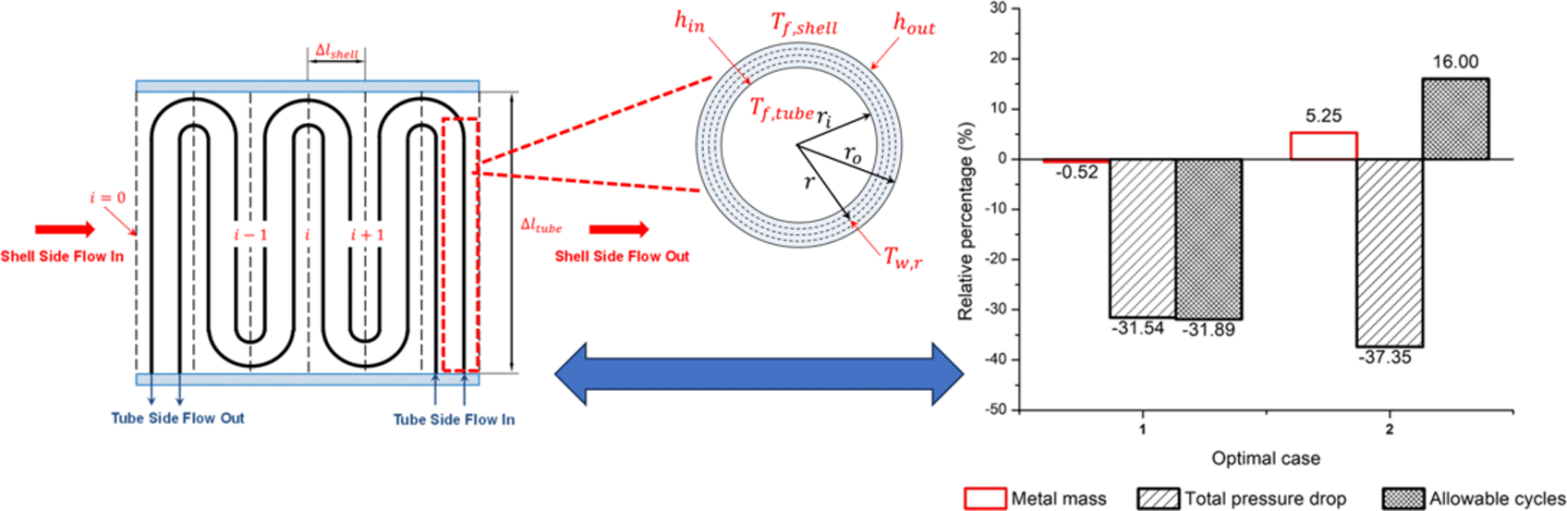

Pulverized coal power plants are increasingly participating in aggressive load-following markets, therefore necessitating the design and optimization of primary superheaters for flexible operations. These superheaters play a critical role in maintaining the final steam temperature of the steam turbine, but their high operating temperatures and pressures make them prone to failure. This study focuses on the optimal design of future-generation primary superheaters for a fast load-following operation. To achieve this, a detailed first-principles model of a primary superheater is developed along with submodels for stress and fatigue damage. Two single-objective optimization problems are solved: one for minimizing metal mass as a measure of capital cost and another for minimizing pressure drop on the steam side as a measure of operating cost. Since these objective functions conflict, a multiobjective optimization problem is executed using a weighted metric methodology. Results from these optimization studies show that the base case design can violate stress constraints during the aggressive load-following operation. However, by optimizing the design variables, it is possible to not only satisfy tight stress constraints but also achieve the desired number of allowable cycles and adhere to the steam outlet temperature constraint. In addition, the optimized design reduces either the metal mass or the steam-side pressure drop compared to that of the base case design. Importantly, this approach is not limited to primary superheaters alone but can also be applied to similar high-temperature heat exchangers in other applications.

## Introduction

Fossil fuel-fired power plants are expected to continue to generate a considerable amount of electricity for the foreseeable future. However, the increasing penetration of renewable energy sources to the grid poses challenges for these power plants, including the need for demanding load-following operations such as fast ramp-up and ramp-down, low-load operation, and frequent start-up and shutdowns. These operational demands affect the health and life span of critical components, especially high-temperature boiler components, often leading to undesired outages and forced shutdowns. One critical requirement for efficient load-following operation by power plants is ensuring the reliability of boiler components. To achieve the desired reliability of these high-temperature boiler components, it is essential not only to optimize the operating conditions but also to design these critical components in an optimal manner for load-following operations. Since most existing boilers are decades old, they were not necessarily originally designed to handle these newer and unforeseen operating challenges.

Flexible operation of energy generation assets is required for improving power plant economics and mitigating CO_2_ emissions.^1−3^ For flexible operation, fast start-ups/shutdowns and steep ramp changes in load must be done more rapidly and more cost-effectively.^4^ The impact of load-following operations on the life consumption of critical steam generation components is not yet fully understood. However, studies have reported that these undesired operations lead to creep and fatigue damages, as well as damages caused by the synergistic effects of creep and fatigue, significantly shortening the life of the boiler components.^5^ Among the critical components, superheaters operating at the highest temperatures are particularly affected by cycling operations. Superheaters not only suffer from fatigue damage but also face the risk of exceeding their design temperature during low-load and cycling operations, leading to creep damages.^6^ It has been reported that superheater damage contributes to about 40% of all boiler unplanned outages,^7^ which, in turn, increases the plant equivalent forced outage rate and/or operation and maintenance cost. Thermal fatigue and creep-fatigue interaction can be considerably damaging and can lead to highly localized damages, such as ligament cracking.^8^ Identification and quantification of the localized damage are challenging without inspection during the plant shutdown. Even with inspections, it can be cost-prohibitive to inspect all possible locations for damage.^5^ One way of reducing the probability of failure occurrences is to optimally design the superheater components for flexible operations. Traditionally superheaters have been designed neither for rapid ramp-up/down operation nor for low-load operations. Therefore, it is highly desirable to design future superheaters while considering creep and fatigue damages resulting from expected increased cycling operations.

While simple models of superheaters are available in the literature,^9^ these models do not account for the complex flow arrangement and heat transfer mechanisms in superheaters. In addition to the convective heat transfer, radiative heat transfer can also take place in superheaters due to the high temperature of the flue gas.^10^ The presence of oxide scale on the inner tube surface and ash accumulation on the exterior of tube surfaces make it difficult to accurately quantify heat transfer resistances along the boiler tubes at any instant of time.^11^ Distributed-parameter dynamic models of superheaters are desirable for capturing the spatial and temporal variabilities of these resistances, as well as the effect of flow arrangement.^12^ A quasi-two-dimensional (2D) radiant pendant superheater model was developed by Rousseau and Gwebu^13^ to quantify the distribution of flows and temperatures on the steam and flue gas sides during ramp changes. Granda et al.^14^ developed a computational fluid dynamics model of a steam superheater to investigate the transient variation of transport profiles due to attemperator activation. However, the works noted above did not evaluate thermo-mechanical stress during transient superheater operation.

During the flexible operation of power plants, the superheaters usually experience elevated temperature gradients and steam pressure variability leading to creep damage and thermo-mechanical fatigue. Madejski and Taler^15^ investigated the temperature and stress distributions in “double-omega” tubes due to the spraying of cold water in the attemperator and during sootblowing for removing ash deposits from superheater tube surfaces. Several authors have studied stress transients in superheater headers.^16,17^ Yasniy et al.^18^ reported that there can be significant damage and multiple cracks due to the nonuniform distribution of temperature through the wall of the headers. Farragher et al.^19^ compared the damage between the inner and outer surfaces and observed that the cracking of the inner side was more pronounced due to steam pressure and steam oxidation. Dynamic modeling and lifetime estimation of boiler components were presented by Benato and co-workers.^20,21^ Their dynamic simulation results showed that a 52.9% reduction of the high-pressure superheater header lifetime was observed when the load change was 50% faster than their reference case while achieving an increase of 35.8% life if the transient was 50% slower. Furthermore, a 7.2% reduction and 10.6% increase in the lifetime of the superheater tube bank were estimated when the equipment ramped 50% faster and 50% slower than the reference case, respectively. In their studies, Benato and co-workers used the dynamic superheater model to predict the tube wall temperatures along both the tube length and the radius. Given the significant temperature transients experienced by the tube wall, particularly during fast load-following operations, it is also crucial to consider the through-wall stress profile to assess damage accumulation accurately.

Dynamic optimization has been widely used for chemical and integrated-energy processes. In a study by Kim et al.,^22^ a MILP-based dynamic optimization was undertaken to optimize dispatch profiles of supercritical pulverized coal (SCPC), natural gas combined cycle (NGCC) plants, and sodium sulfur batteries for varying renewable penetration levels. Pattison et al.^23^ proposed an approach for optimal scheduling of the continuous processes subjected to high market variability. Dering et al.^24^ presented a dynamic optimization approach for the basic oxygen furnace leading to a 6.7% decrease in the amount of carbon emission and a 2.9% decrease in the production cost compared to the base case. Yancy-Caballero et al.^25^ undertook constrained optimization for the oxidative coupling of methane to improve C_2_H_4_ and C_2_H_6_ yield. Leipold et al.^26^ focused on optimizing the periodic operation of methanol synthesis in an isobaric and isothermal fixed-bed reactor. Bremer et al.^27^ undertook dynamic optimization for a fixed-bed reactor used for CO_2_ methanation for determining control trajectories that can prevent distinct hot spot formation during a time-optimal start-up.

Similar to chemical and process plants, dynamic optimization can be applied to improve the operational efficiency of power plants, especially under load-following operation.^1^ Quantifying thermo-mechanical stresses for plant equipment and including them in dynamic optimization as constraints can help to reduce damage of critical assets. A comprehensive review of the literature on dynamic power plant modeling and optimization can be found in Alobaid et al.^28^ Kruger et al.^29^ developed the optimum start-up strategy without violating the stress limitation for both drum and superheater outlet headers in power plant boilers. Kim et al.^30^ analyzed the thermal stress evolution in the steam drum of a heat-recovery steam generator under three different start-up strategies. Rúa et al.^31,32^ developed model predictive control (MPC) to ensure that the stress constraints in the steam drum and the turbine rotor in an NGCC plant were not violated during plant transients. In our previous study,^33^ dynamic optimization was undertaken for maximizing the efficiency of a coal-fired power plant while maintaining the main steam temperature during transient operation. A comprehensive dynamic optimization study was conducted by some of the authors of this paper^5^ for load-following operation of an NGCC plant under stress constraints for the steam drum. It was observed that for certain desired ramp rates and stress constraints, satisfying the stress constraints is not feasible without relaxing the ramp rate. Therefore, multiobjective dynamic optimization was undertaken for minimizing ramp rate relaxation while maximizing plant efficiency under stress constraints. However, that study optimized only plant operating conditions and focused only on the steam drum.

To the best of our knowledge, there is currently no work in the literature on the optimal design and operation of superheaters under transient operations with considerations of thermal stress and fatigue damage. Optimal superheater design is critical for achieving higher power plant performance since superheaters operate at elevated temperatures and need to transfer large amounts of heat without causing a greater pressure drop than desired (only a few psi). Minimizing metal mass can be considered as an objective function for superheater design.^34,35^ Less metal mass not only reduces capital cost but also enables lower thermal hold-up and faster transients, making the superheater more desirable for fast load-following operations. In addition, a lower pressure drop through the superheater tubes leads to higher-pressure steam entering the steam turbine, thereby increasing the power production. Thus, it is desirable to minimize the pressure drop as a measure of the operating cost. However, the optimization objectives mentioned above are conflicting; therefore, a multiobjective optimization problem is solved in this work for optimal design of the superheater.

## Superheater Configuration and Model

**Figure 1:**
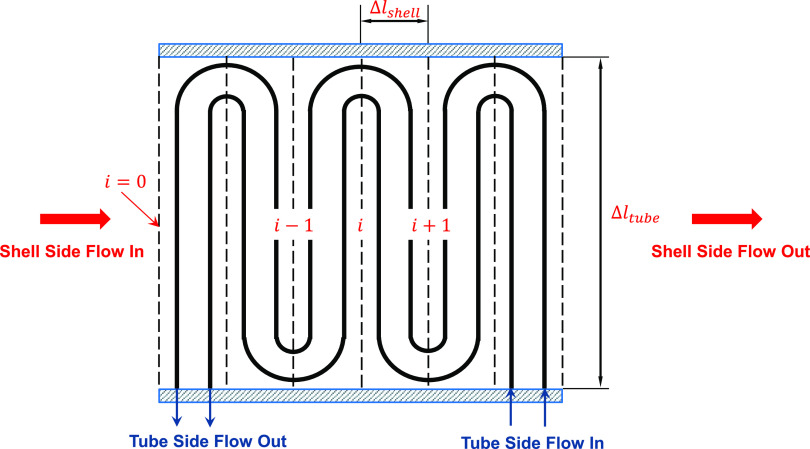
Schematic diagram of a typical superheater and discretization along the flow path of the shell-side fluid.

A superheater is a cross-flow shell-and-tube heat exchanger where the hot flue gas flows on the shell side, while the cold steam flows inside the tubes. Because the fluid temperature changes along the flow path on both the shell and tube sides, the velocity of the fluid also changes from the inlet to the outlet. There is also spatial variation of parameters such as the heat transfer coefficients and friction factors on both the shell and tube sides. To model the spatial variation of transport variables, a two-dimensional (2D) model is constructed with discretization along the flow path and across the tube thickness. Figure 1 illustrates the schematic diagram of the typical superheater with a parallel-serpentine tube bundle configuration that is modeled in this paper. The steam enters the tubes of multiple parallel rows with each row containing multiple columns to form a tube bundle. For simplicity, as seen in Figure 1, the superheater configuration shows just two tube rows, but a bundle with 2–8 tube rows is very common.

Due to the high metal mass and heat capacity of the tube wall, its thermal holdup should not be ignored especially for a dynamic superheater model intended for simulating transient processes during load-following operations. Therefore, in addition to modeling the temperature profiles of steam and flue gas along their flow paths, spatial and temporal variations of the temperature along the tube wall thickness need to be modeled as well. Evaluation of the spatiotemporal temperature profiles is particularly important for a model intended for studying local stress evolution and its impact on equipment health. Those considerations lead to the development of a 2D partial differential equation (PDE) first-principles model. Although the flue gas flow direction and the overall steam flow direction are perpendicular to one another, the energy balances are still one-dimensional for each fluid.^36^ The energy conservation for the flue gas side is given by eq 1
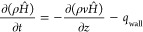
1In eq 1, ρ,*v*, and *Ĥ* denote the density, velocity, and specific enthalpy of the flue gas, respectively, and *q*_wall_ denotes the heat loss from the flue gas to the shell-side boundary of the tube wall. Note that for most engineering applications, the convective term (the first term on the right side of the equation) is much larger than the conduction term, and hence the conduction term is ignored in the energy equation. A similar equation is written for the steam side. Both the flue gas and steam sides are discretized. The shell-side flow is divided into multiple segments as shown in Figure 1. Due to the parallel-serpentine-tube configuration, the flow direction inside the tube is altered between two neighboring tube segments. Flow properties are calculated based on the transport variables, as appropriate, in individual discrete elements.

**Figure 2:**
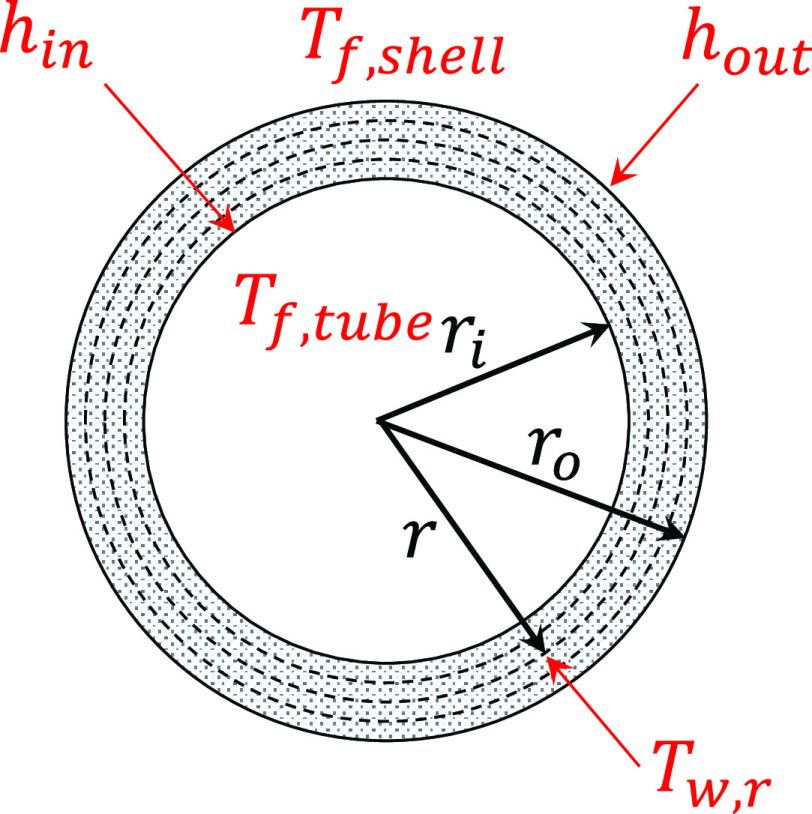
Schematic diagram showing the discretization of tube wall temperature along the tube radius and various variables for the tube wall model.

Figure 2 illustrates the discretization of the tube wall temperature along the tube radius direction. The tube wall temperature *T*_wall*,r*_ at radius *r* is calculated based on the transient heat conduction equation as shown below
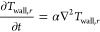
2where α is the thermal diffusivity of the tube material.

The boundary condition at the inner tube wall, i.e., *r* = *r*_*i*_, is given by
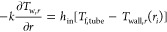
3where *k* is the thermal conductivity of the tube metal, *T*_f,tube_ is the temperature of the fluid inside the tube, and *h*_in_ is the equivalent convective heat transfer coefficient between the tube inner wall and the steam while accounting for the heat transfer resistance due to fouling.

The boundary condition at the outer tube wall, i.e., *r* = *r*_o_, is given by
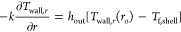
4where *T*_f,shell_ is the shell-side flue gas temperature and *h*_out_ is the equivalent heat transfer coefficient by considering the convective heat transfer, radiative heat transfer, if any, and the fouling resistance on the shell side.

The convective heat transfer coefficient *h*_in,conv_ on the tube side is calculated as follows
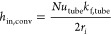
5where *k*_f,tube_ is the thermal conductivity of the fluid inside the tube. The Nusselt number, *Nu*_tube_, for turbulent flow in a tube is calculated by relating it to the Reynolds number *Re*_tube_ and Prandtl number *Pr*_tube_(37) as follows
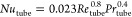
6

The Reynolds number is given by
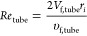
7where υ_f,tube_ is the kinematic viscosity of the fluid inside the tube and *V*_f,tube_ is the fluid velocity.

*h*_in_ is calculated by
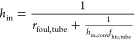
8where *f*_htc,tube_ is a correction factor for heat transfer coefficient due to a nonuniform distribution of the flow inside multiple tubes and *r*_foul,tube_ denotes the fouling resistance at the inside of tubes. If the tube-side distribution is uniform, then *f*_htc,tube_ = 1

*h*_out,conv_ is calculated using
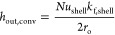
9where *k*_f,shell_ is the thermal conductivity of the fluid on the shell side and *Nu*_shell_ is the shell-side Nusselt number. The following equation is used for calculating *Nu*_shell_(38)
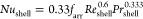
10where *Re*_shell_ and *Pr*_shell_ are the shell-side Reynolds number and Prandtl number, respectively, and *f*_arr_ is a factor that depends on the tube arrangement. For the staggered tube arrangement, *f*_arr_ = 1, and for the in-line tube arrangement, *f*_arr_ = 0.788.

*Re*_shell_ is calculated as follows
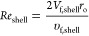
11where υ_f,shell_ is the kinematic viscosity of the fluid on the shell side and *V*_f,shell_ is the flue gas velocity on the shell side.

When the temperature of the fluid on the shell side is significantly high (i.e., above 800 K), heat transfer through radiation on the shell side must be considered. An algebraic surrogate model for calculating gas emissivity is developed using ALAMO^39^ from a narrow band high-fidelity model RADCAL, developed at the National Institute of Standards and Technology (NIST).^40^ The gas emissivity is expressed as a function of the mole fractions of CO_2_, H_2_O, O_2_, and N_2_, the pressure on the shell side, and the mean beam length estimated based on the tube outside diameter and pitches between neighboring tubes.^41^

The mean beam length is calculated based on the ratio of volume to surface area of the shell-side cavity^42^
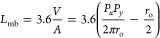
12where *P*_*x*_ and *P*_*y*_ are the pitches (distance between two neighboring tubes) in the parallel and perpendicular directions to the shell-side flow, respectively.

The gas-surface radiation exchange factor *F*_rad_ for the shell-side wall is calculated as^43^
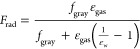
13where *f*_gray_ is the fraction of gray gas in the entire spectrum, which is calculated from the gas emissivity surrogate model, ε_gas_ is the gas emissivity at the shell-side fluid temperature, and ε_w_ is the emissivity of the tube wall considering any fouling. The radiation contribution to the shell-side heat transfer coefficient after linearizing the radiation heat transfer rate expression is^43^
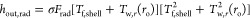
14where σ is the Stefan–Boltzmann constant.

Finally, by considering the fouling resistance on the shell side *r*_foul,shell_, the equivalent heat transfer coefficient on the shell side is calculated as
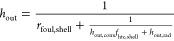
15where *f*_htc,shell_ is a correction factor for convective heat transfer coefficient due to nonuniform flow distribution on the shell side. *f*_htc,shell_ = 1 for a uniformly distributed flow.

For calculating the tube-side pressure drop, the Darcy friction factor is calculated based on the turbulent flow^37^
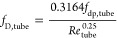
16where *f*_dp,tube_ is a correction factor accounting for the roughness of the tubes. The pressure drop per unit length of the tube is calculated as
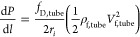
17where ρ_f,tube_ is the fluid density on the tube side.

For the staggered tube arrangement, the friction factor for the shell side is calculated by^44^
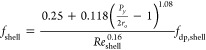
18where *P*_*y*_ is the pitch in the direction perpendicular to the shell-side flow and *f*_dp,shell_ is the correction factor due to nonuniform flow distribution on the shell side.

For in-line tube arrangement, the friction factor is calculated as^44^
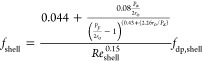
19where *P*_*x*_ and *P*_*y*_ are the pitches in the parallel and perpendicular directions to the shell-side flow, respectively.

The pressure drop for each tube row is calculated as^44^
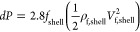
20where ρ_f,shell_ is the fluid density on the shell side.

## Models for Superheater Stress and Fatigue Damage

### Stress Model

Using the classic elasticity theory,^45^ thermo-mechanical principal stresses are calculated for a radial tube location *r*_*i*_. For computing principal mechanical stresses in the radial, tangential, and axial directions, the following equations are used, respectively^46^
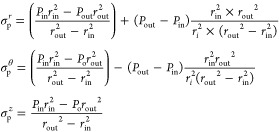
21where *P*_in_, *r*_in_ and *P*_out_, *r*_out_ are the pressure and radius at the inside and outside surfaces, respectively.

Thermal stresses are computed using the following equations^47,48^
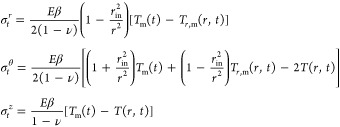
22where *E* is the Young’s modulus, β is a linear temperature expansion coefficient, and ν is the Poisson’s ratio of the material of construction.

The mean temperatures *T*_m_ and *T*_*r*,m_ are calculated using the following equations
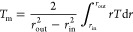
23
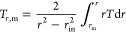
24In this study, the integrals in eqs 23 and 24 are calculated by using the trapezoidal rule.

The von Mises stress, which is a scalar measure capturing the overall effect of the principal stress components, is computed as follows
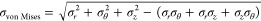
25where σ*_r_*, σ_θ_, and σ*_z_* are the principal structural stresses including mechanical and thermal stresses that are calculated as follows
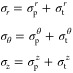
26

### Fatigue Damage Model

The maximum number of allowable cycles is evaluated using the international standard EN 13445.^46^ The allowable number of fatigue cycles *N* is computed as
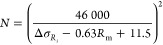
27where *R*_m_ is the material tensile strength at room temperature. The reference stress range Δσ*_R_i__* depends on the stress range Δσ_*i*_ and is calculated as follows
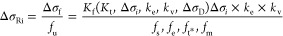
28where *K*_f_ indicates the effective stress concentration factor. *K*_f_ depends on the theoretical stress concentration factor *K*_t_, the stress range Δσ_Ri_, the plasticity correction factors *k*_e_ and *k*_v_, and the material endurance limit Δσ_D_.

The overall correction factor *f*_u_ is dependent on the surface finish correction factor (*f*_s_), the thickness correction factor (*f*_e_), the temperature correction factor (*f*_t*_), and the mean stress correction factor (*f*_m_). Because the reference stress range Δσ_Ri_ depends on N and N is computed as a function of Δσ_Ri_, an iterative calculation is used to determine N.

### Dynamic Optimization

The dynamic optimization of a superheater in a subcritical pulverized coal power plant is undertaken to minimize the metal mass and/or the total pressure drop while satisfying the outlet steam temperature and the equipment stress constraint under load-following operation. In this study, a load ramp rate of 10% is evaluated, which is about the maximum rate desired in a power plant operation. For the dynamic optimization, the load is ramped down from 100, to 50% and back up to 100%. The design variables optimized for improved performance include tube thickness, tube inner diameter, tube length per segment, number of tubes in each row, and number of inlet tube rows. Single-objective optimization problems are initially solved, followed by subsequent solution of a multiobjective optimization.

### Single-Objective Optimization

#### Single-Objective Optimization of Metal Mass

One of the single-objective optimizations is to minimize the metal mass, *M*_metal_tube_. The optimization problem is given in eq 29. It should be noted that while the normalization of the metal mass as given in the objective function *J*_1_ is not necessary for the single-objective optimization, it is done mainly for the multiobjective optimization.
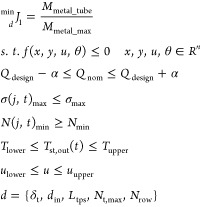
29The first constraint includes the equality and inequality constraints related to the superheater process models and stress and fatigue models described before. The second constraint ensures that the heat duty of the superheater under nominal conditions, *Q*_nom_, remains within the allowable heat duty deviation α. The third constraint bounds the stress, σ, at the location with the highest stress below the upper bound σ_max_ by considering the entire temporal profile. The fourth constraint bounds the allowable number of cycles *N* at the location, with the minimum number of allowable cycles to be higher than the desired minimum number of cycles by considering the entire temporal trajectory. It should be noted that satisfying the third and fourth constraints is challenging since neither the location nor the time of the maximum stress and minimum number of allowable cycles is known a priori. In addition, both location and time can vary as the design variables are optimized. The fifth constraint ensures that the outlet steam temperature remains bounded during the entire operation. It should be noted that, like the third and fourth constraints, satisfying the fifth constraint is challenging due to the uncertainty of when the outlet steam temperature may potentially violate the temperature constraint. Finally, the sixth constraint keeps the decision variables bounded.

#### Single-Objective Optimization of Pressure Drop

Another single-objective optimization is to minimize the integral pressure drop through the tube (i.e., the steam side) over the entire time of operation. The optimization problem is given by eq 30, where Δ*P*_total_ is the instantaneous pressure drop. Constraints are the same as those for the previous single-objective optimization.
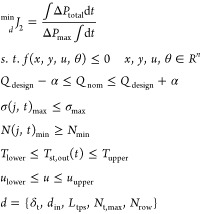
30

#### Multiobjective Optimization

For the multiobjective optimization, a weighted metric approach is used. A general formulation is given below
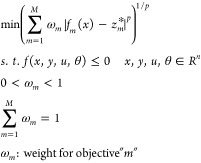
31If the minimization problem is completely convex, provided that the weights ω_*i*_ > 0, any solution found using the weighted metric technique is a Pareto-optimal solution.

For this problem, there are two weighting factors, ω_1_ and ω_2_. The value of ω_1_ is varied from 0.1 to 0.9 with an interval of 0.1. *p* is the metric used to measure the distance between the reference point and the objective feasible region, and it is set to 2. The resulting multiobjective optimization problem is given by
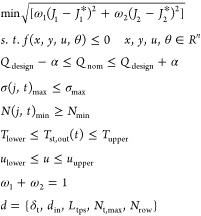
32In eq 32, optimal values of the objective functions from the single-objective optimizations described above are denoted by *J*_1_^*^ and *J*_2_^*^, respectively.

**Figure 3:**
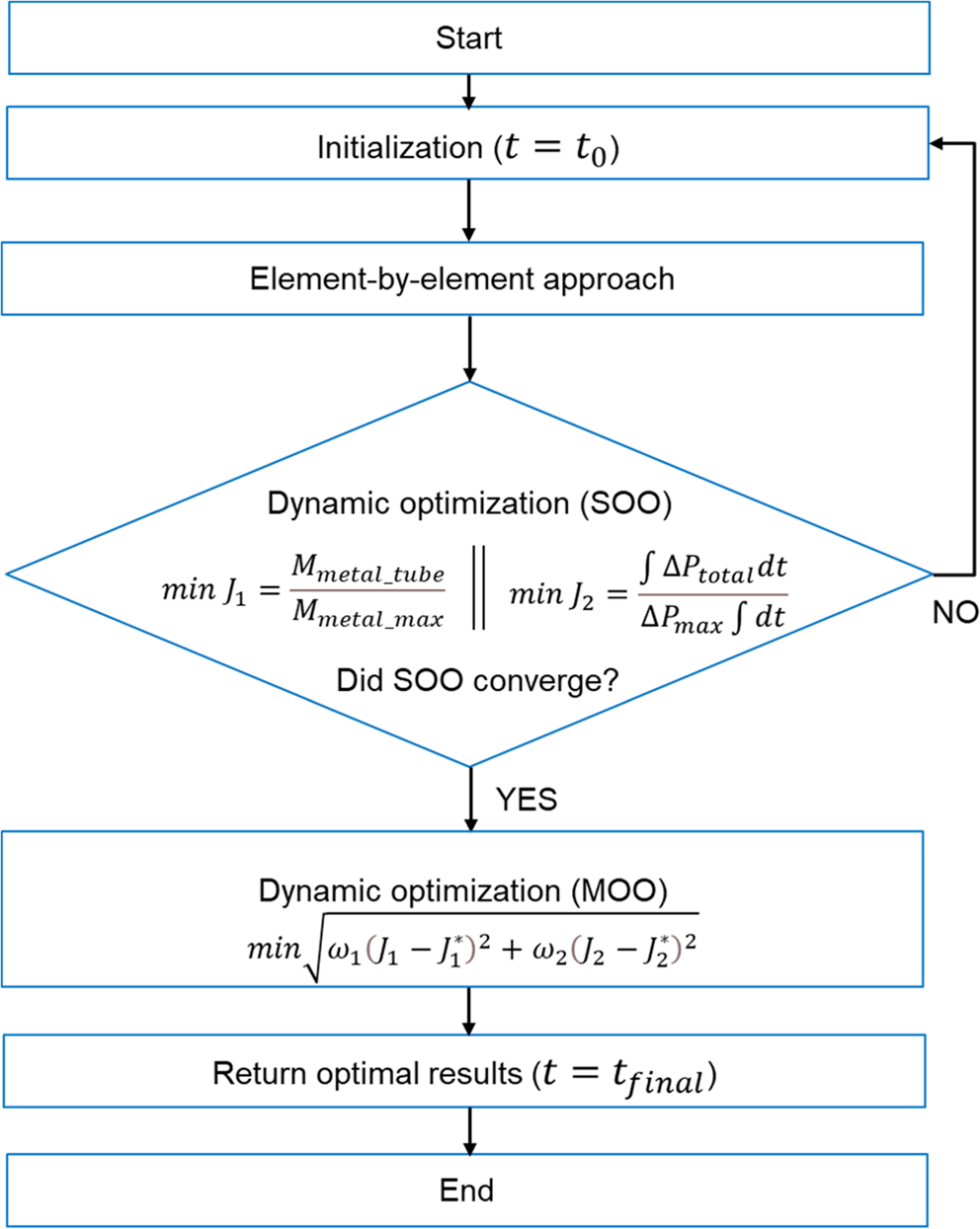
Workflow for the dynamic optimization of the primary superheater.

In summary, Figure 3 illustrates a general procedure for solving the multiobjective dynamic optimization in this study. The time domain is fully discretized^49^ to facilitate satisfying the constraints for stress, minimum number of allowable cycles, and the main steam temperature. However, one of the difficulties when solving the fully discretized problem is the generation of good initial guesses for all variables as the decision variables are changed. If the single-variable optimization problems fail to converge for given values of design variables, then the initialization problem is resolved and an element-by-element approach is employed to simulate the model for the entire time range. The element-by-element solution approach is similar to the typical dynamic integrator approach but adapted for a fully discretized approach. In this approach, the entire time range is divided into multiple time windows, and only 1 time window is solved at a time. The values of all variables at the end of a window are used not only for initializing the appropriate variables for the next window but also as the initial guess for all variables for the next entire time window. This continued until the end of the time range is reached. The element-by-element approach is found to be highly effective in reliably solving single-variable optimizations.

### Design Constraints and Optimization

Table 1 lists the base case values and bounds for the decision variables for the design of the primary superheater.

**Table 1:** Base Case Values and Bounds for the Design Decision Variables

decision variables	unit	base case	lower bound	upper bound
tube thickness (δ_*t*_)	mm	3.8100	3.2385	4.3815
tube inner diameter (*d*_in_)	mm	36.8300	31.3055	42.3545
tube length per segment (*L*_tps_)	m	7.6835	6.852	8.4519
maximum number of tubes in a row (surrogate variables for the max width) (*N*_*t*,max_)	-	88	44	104
number of inlet tube rows (*N*_row_)	-	4	2	5

## Results and Discussion

### Simulation of an Existing Superheater

The process model for the primary superheater is developed in the Pyomo-based IDAES software.^50^ IAPWS-95 is used for the steam properties.^51^ PYOMO.DAE is used for discretizing the differential equations.^52^ The fully discretized model yields a set of nonlinear algebraic equations, which is solved using IPOPT.^53^

Tables 2 and 3 list the base case specifications and boundary conditions for the superheater, respectively. The base case conditions, specifications, and boundary conditions listed in Tables Tables 1–3, respectively, are the same as those used in our earlier work.^33^

**Table 2:** Base Case Specifications of the Superheater

	unit	specifications
flow type		counter-current
tube arrangement		in-line
tube pitch between rows (i.e., longitudinal pitch parallel to the flue gas flow direction)	mm	95.25
tube pitch between columns (i.e., longitudinal pitch perpendicular to the flue gas flow direction)	mm	152.4
number of passes		9
material of construction (MOC)		SA209 T1
density of MOC	kg/m^3^	7800
elevation change from inlet to outlet	m	5.0

**Table 3:** Steam and Flue Gas Boundary Conditions

	unit	value
flue gas inlet temperature	K	1195.2
steam inlet temperature	K	610.5
flue gas inlet pressure	bar	0.85
steam inlet pressure	bar	133.62
flue gas flow rate	mol/s	9551.85
steam flow rate	mol/s	11471.71

**Figure 4:**
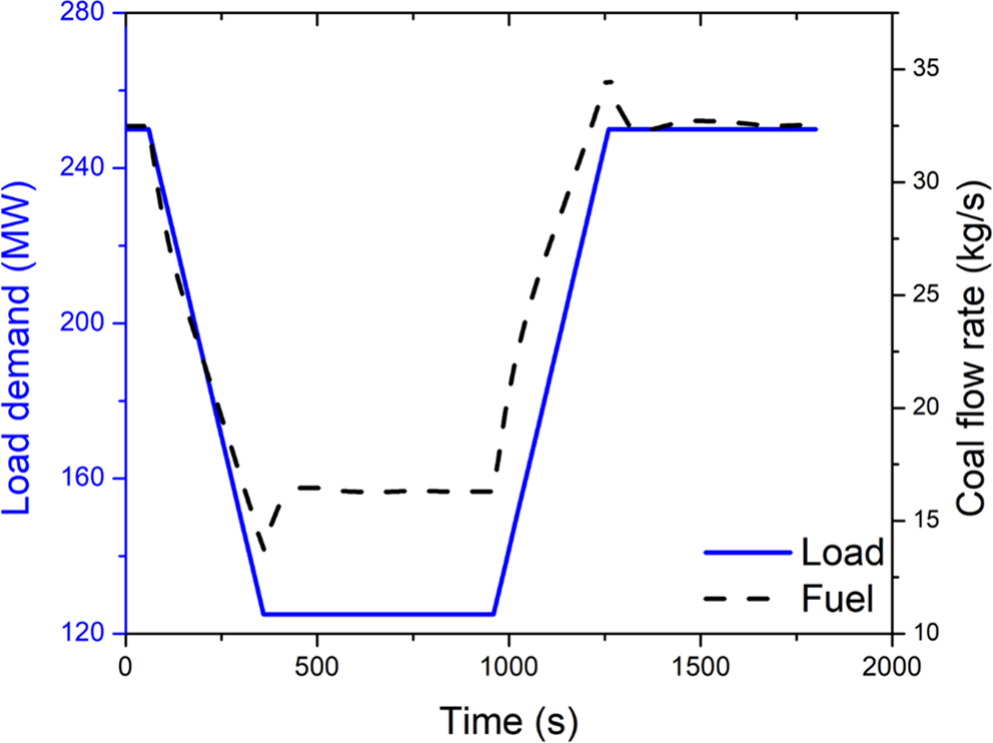
Transient response for the simulated dynamics in load.

Figure 4 shows the dynamic simulation of two load changes at ramp rates of 10%/min. The dynamic model of the power plant used for simulating the load change has been presented in our earlier works.^33,51^ The load is changed from the full load to 50% and maintained at 50% load for 10 min before ramping back up to the full load. Figure 4 also shows the corresponding change in the coal flow rate. The undershoot in the coal flow rate at the end of the downward ramp is due to the release of stored thermal energy in the boiler, mainly from the drum and waterwalls, and the overshoot above the steady-state value at the end of the upward ramp is because extra fuel is fired to build up the stored energy. It is obvious that the flue gas flow rate will have characteristics similar to those of the coal flow rate.

**Figure 5:**
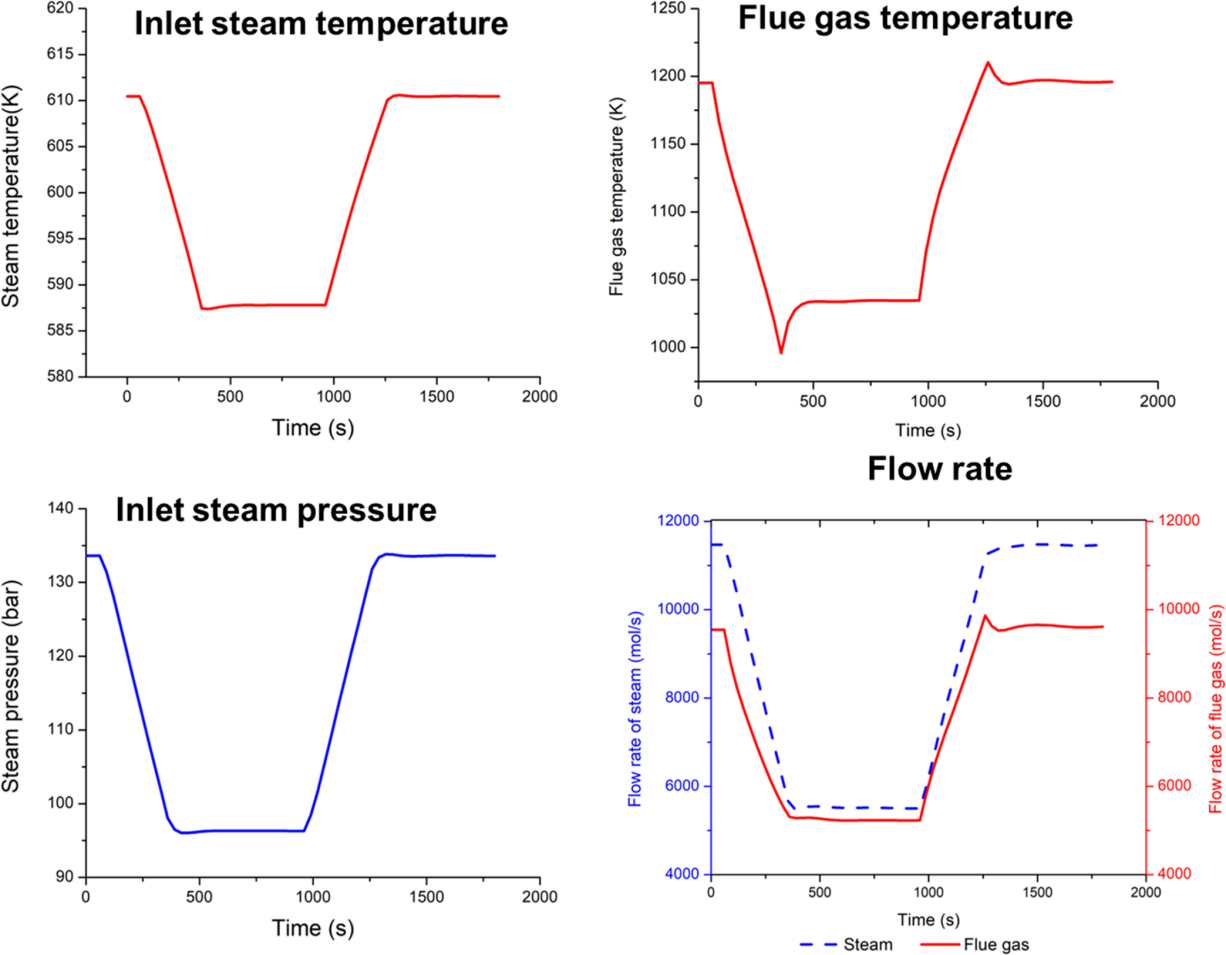
Transient response in steam and flue gas conditions at the inlet of the primary superheater.

**Figure 6:**
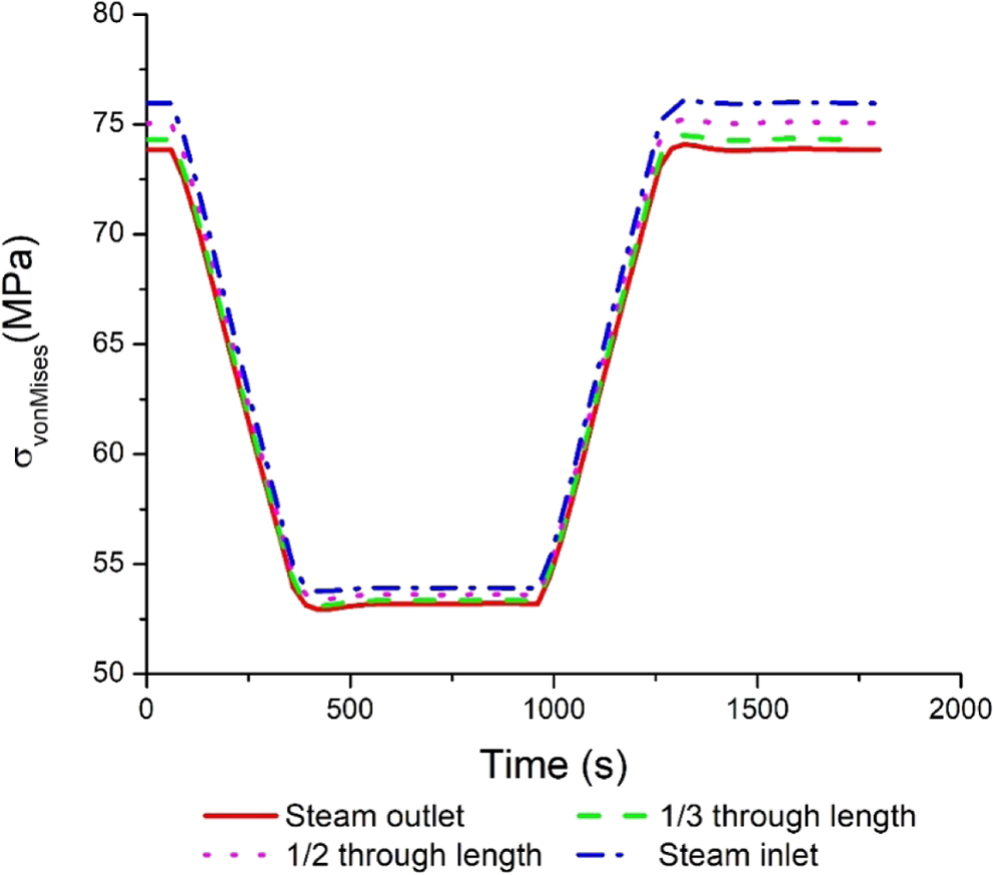
Transient response in the von Mises stress distribution profiles at different locations in the superheater tube.

Figure 5 presents the transient response in the superheater inlet steam temperature and pressure, flue gas temperature, and flow rates of the steam and flue gas. The undershoot and overshoot in the flue gas temperatures correspond to the similar undershoot and overshoot in coal flow rates. Figure 6 presents the distributed profiles of the von Mises stress in the superheater. The highest stress is found to occur at the steam inlet location.

### Impact of Different Ramp Rates on Fatigue and Creep Damages

**Figure 7:**
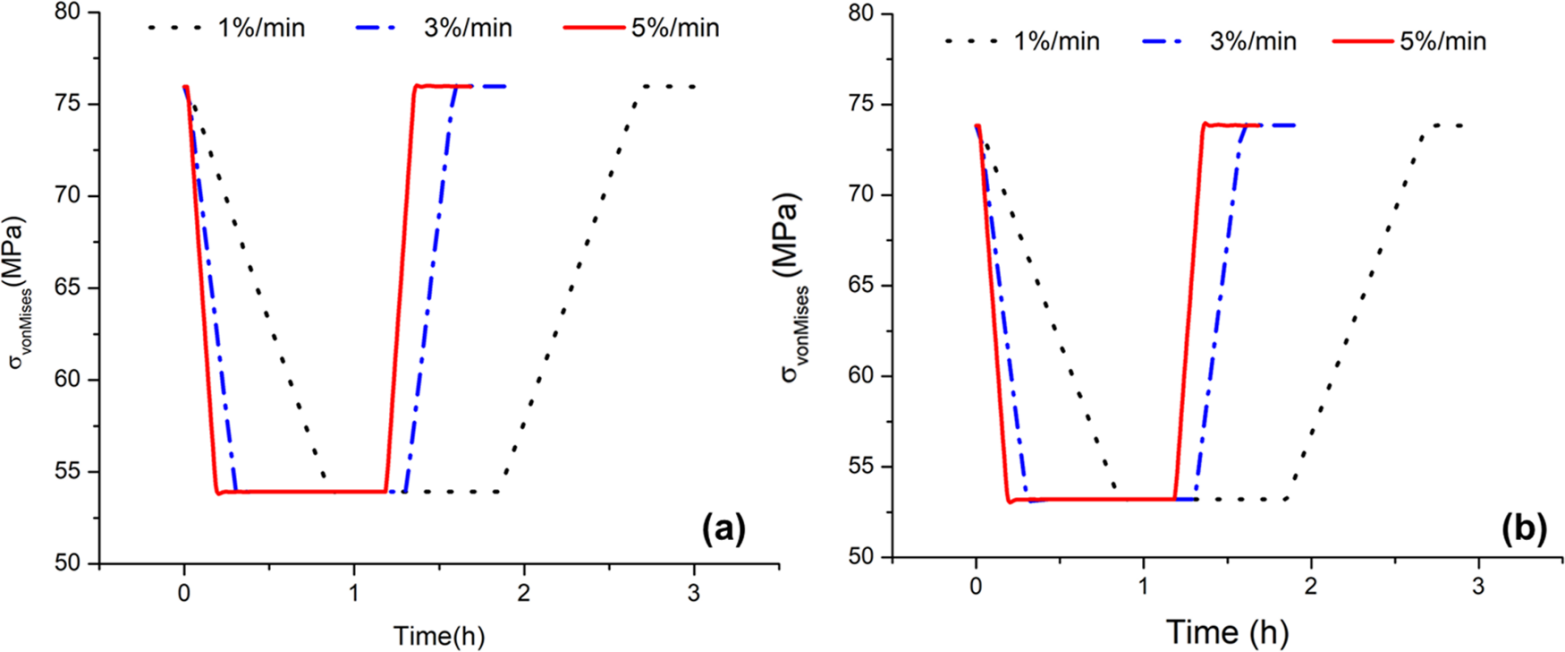
Sensitivity analysis of different ramp rates on total Von Mises stress at the (a) steam inlet location and (b) steam outlet location.

**Figure 8:**
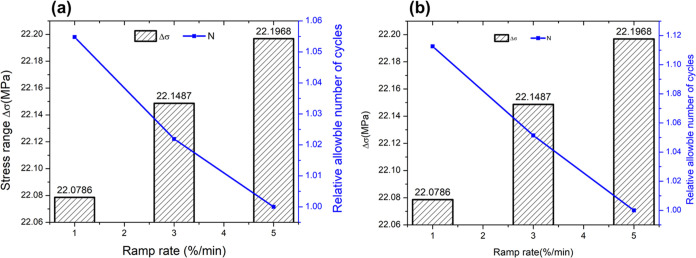
Impact of different ramp rates on fatigue damage on (a) steam inlet location and (b) steam outlet location.

The impacts of ramp rates on superheater fatigue and creep damages are studied by simulating 1, 3, and 5%/min ramp changes in load with the load variation as in Ma et al.^33^Figure 7 presents the effect of different ramp rates on the total Von Mises stress at the steam inlet and outlet locations. The stress remains slightly higher at the inlet location. It is found that while the rate of change of the stress does differ with the ramp rate as expected, the highest and lowest values of stress do not differ with the ramp rate. Figure 8 shows the change in the fatigue damage, including the relative allowable number of cycles at the steam inlet and outlet locations. The relative allowable number of cycles is calculated by scaling the allowable number of cycles for any given ramp rate with respect to the allowable number of cycles at a 5%/min ramp rate. It is found that at the steam inlet location, a ramp rate of 1%/min results in approximately 5% more allowable cycles compared to a ramp rate of 5%/min. At the steam outlet location, a ramp rate of 1%/min yields approximately 11% more allowable cycles than a ramp rate of 5%/min.

### Optimization Results

#### Single-Objective Optimization of Metal Mass

For the single-objective optimization that seeks to minimize the metal mass, two cases are considered where the only difference is in the constraint for maximum stress, i.e., σ_max_. The value of σ_max_ can vary significantly depending on the material of construction, desired lifetime, expected operational profile, level of conservatism by the utility, etc. Based on sensitivity studies, it was found that the maximum stress reached at any location at any time instant remains below 80 MPa. Therefore, in Case 1, the σ_*max*_ value is set to 150 MPa, significantly higher than the maximum stress experienced at any location or time instant, serving as a case where the stress constraint remains inactive. In Case 2, σ_max_ is set to be 75 MPa, making the stress constraint active for both single-objective and multiobjective optimizations. For both cases, the fatigue damage constraint *N*_min_ is set to 0.57*N*_min_^*^, where *N*_min_^*^ is the minimum number of allowable cycles obtained in the base case simulation.

Table 4 lists the results for the superheater design variables for the unconstrained (Case 1) and constrained (Case 2) optimizations as well as the base case values from Table 1. The optimized tube thickness is slightly higher for Case 2 compared to Case 1 since the stress constraint is tighter for Case 2. However, the tube thickness for both optimization cases is still lower than the thickness in the base case. Tube diameter shows the opposite trend and keeps decreasing as the stress constraint is tightened. Tube length for both Cases 1 and 2 does not change but is found to be lower than the base case. The maximum number of tubes in a row for Cases 1 and 2 does not change but is found to be higher than the base case.

**Table 4:** Optimal Design of Primary Superheater for the Single-Objective Optimization of Metal Mass

case	unit	base case	case 1	case 2
tube thickness	mm	3.81	3.61	3.63
tube diameter	mm	36.83	34.89	34.40
tube length	m	7.68	6.85	6.85
maximum number of tubes in a row (surrogate variables for the max width)		88	96	96
number of inlet tube rows		4	4	4

**Figure 9:**
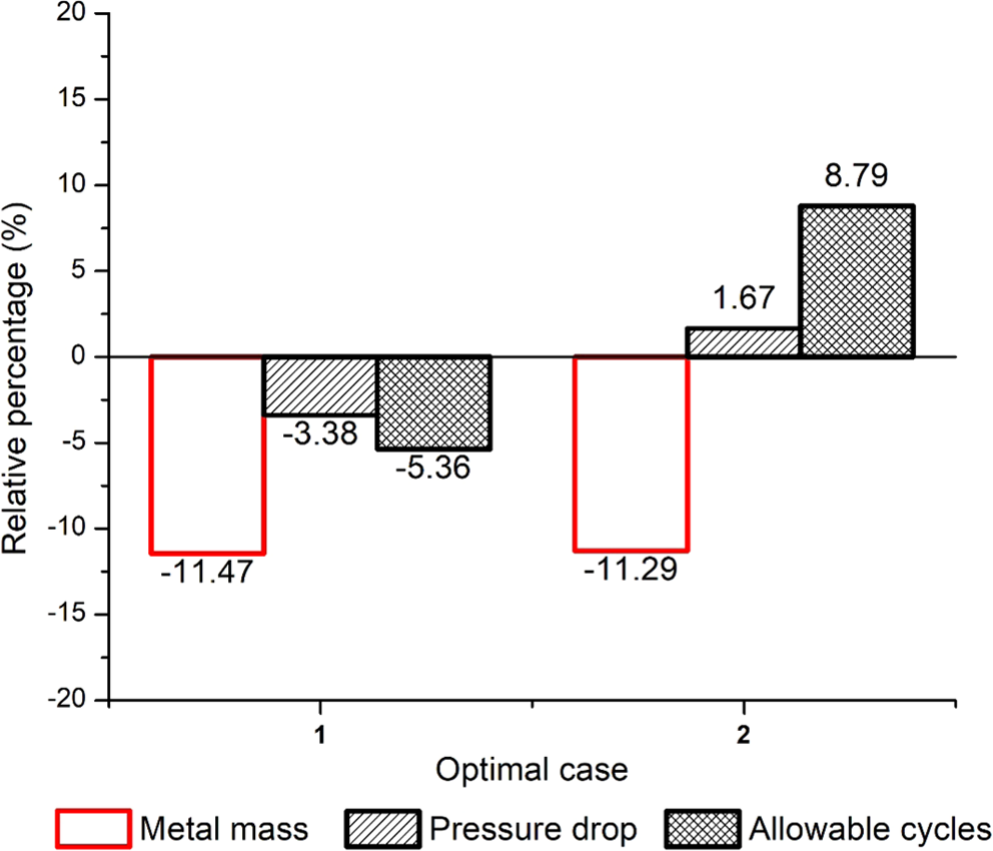
Percentage change in metal mass, pressure drop, and allowable number of cycles for Cases 1 and 2 with respect to the base case for the single-objective optimization of metal mass.

Figure 9 shows the % change in the metal mass, pressure drop, and the allowable number of cycles for Cases 1 and 2 with respect to the base case. In Figure 9, a negative value indicates that the value of the corresponding variable is lower than that in the base case. The required metal mass is smaller for both Case 1 and 2 compared to the base case. However, the metal mass is reduced more in Case 1 since the maximum stress is unconstrained. In Case 1, the pressure drop and allowable number of cycles are lower than the base case, while the opposite trend is observed for Case 2. The transient profile of the steam outlet temperature, maximum stress, and pressure drop for Case 2 versus the base case can be found in the Supporting Information in Figures S1.1–S1.3. As expected, the steam outlet temperature constraint is always satisfied. It is also observed that there is a considerable transient change in the maximum stress mainly because of the transient change in the steam pressure.

#### Single-Objective Optimization of Pressure Drop

Table 5 lists the results for the superheater design variables when minimizing pressure drop for the same inactive stress (Case 1) and constrained active stress (Case 2) cases used in the previous single-objective optimization, respectively. Tube thickness in Case 2 is higher than Case 1 as the stress constraint is tightened like in single-objective optimization of metal mass; however, unlike in the single-objective optimization of metal mass, tube thickness for both Cases 1 and 2 is higher than that in the base case. Tube diameter remains the same for both Cases 1 and 2 and is about 10% higher than the base case, as would be expected. These results are in contrast to the single-objective optimization of metal mass, where the tube diameter was 5.3 and 6.6% lower for Cases 1 and 2, respectively, compared to the base case. Tube length for both Cases 1 and 2 does not change but is found to be lower than that of the base case and at the lower bound as in the single-objective optimization of metal mass. The maximum number of tubes in a row for Cases 1 and 2 does not change but is found to be lower than the base case, unlike in single-objective optimization of metal mass. Overall, these results show the opposing trends obtained in the two single-objective optimizations for tube thickness, tube diameter, and the maximum number of tubes in a row.

**Table 5:** Optimal Design of the Superheater for the Single-Objective Optimization of Pressure Drop

case	unit	base case	case 1	case 2
tube thickness	mm	3.81	4.22	4.31
tube diameter	mm	36.83	40.51	40.51
tube length	m	7.68	6.85	6.85
maximum number of tubes in a row (surrogate variables for the max width)		88	80	80
number of inlet tube rows		4	4	4

**Figure 10:**
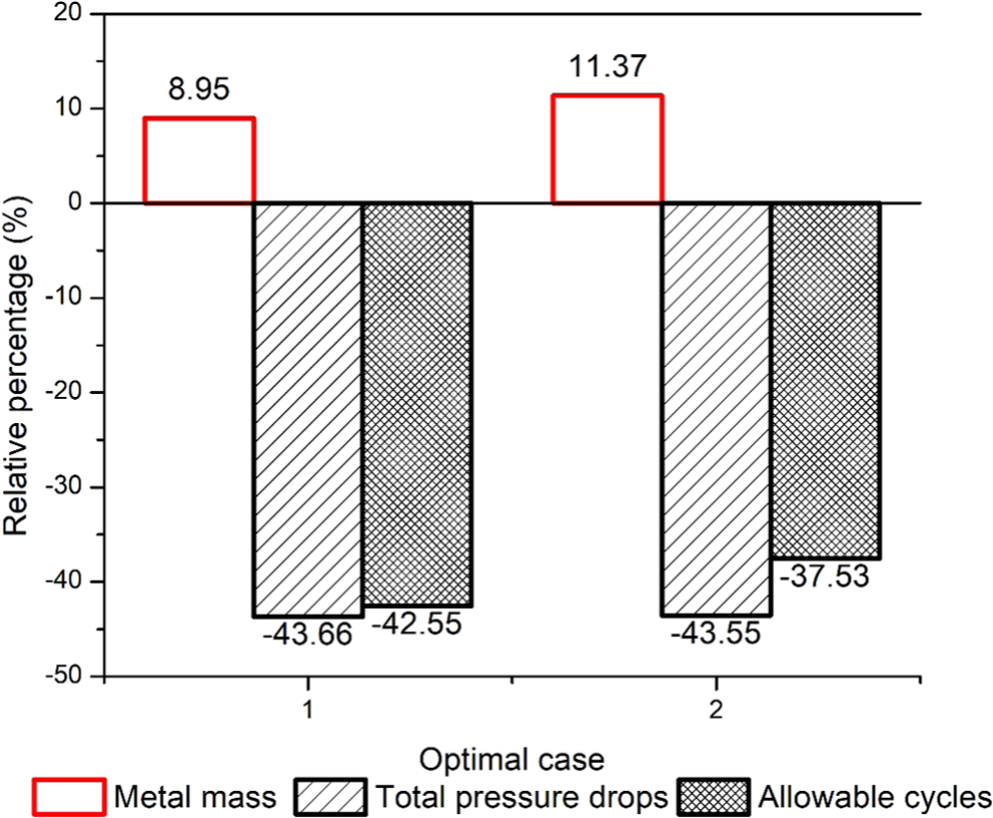
Percentage change in metal mass, pressure drop, and allowable number of cycles for Cases 1 and 2 with respect to the base case for the single-objective optimization of pressure drop.

Figure 10 shows the % change in the metal mass, pressure drops, and the allowable number of cycles for Cases 1–2 with respect to the base case. In contrast to the single-objective optimization of metal mass, the pressure drop is much lower than the base case and the single-objective optimization of metal mass, as would be expected. The metal mass is higher for both Cases 1 and 2 compared to the base case and the single-objective optimization of the metal mass. For both Cases 1 and 2, the allowable number of cycles is lower than the base case and obviously much lower than Cases 1 and 2 for the single-objective optimization of metal mass. In general, these results show the conflict between the objective functions for metal mass and pressure drop minimization, thus motivating the multiobjective optimization. Transient profiles of the steam outlet temperature, maximum stress, and pressure drop for the base case versus Case 2 can be found in the Supporting Information in Figures S1.4–S1.6.

#### Multiobjective Optimization

**Figure 11:**
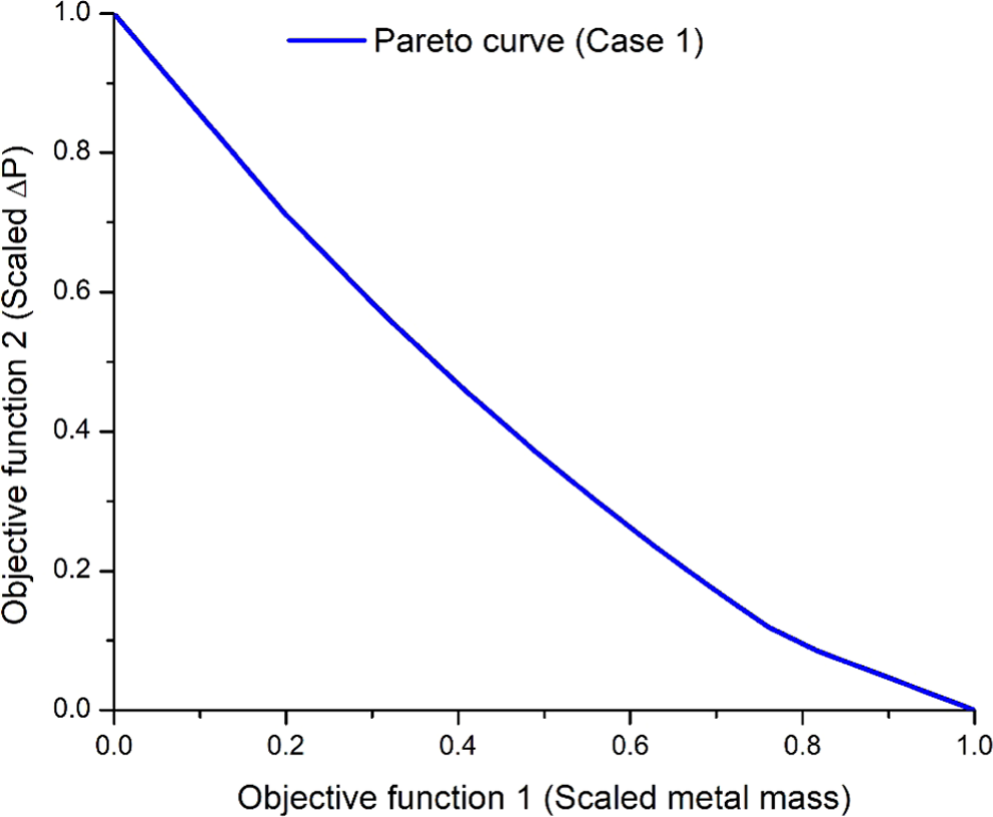
Pareto curve for multiobjective optimization for the inactive stress constraint case (Case 1).

**Figure 12:**
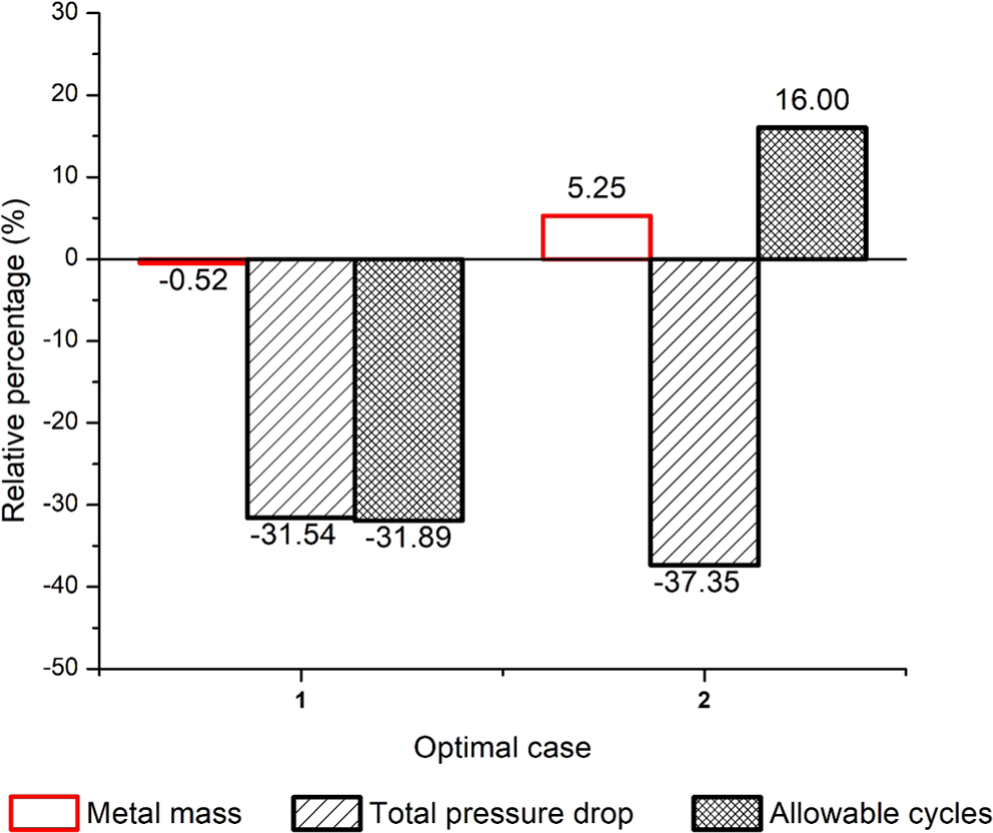
Percentage change in metal mass, pressure drop, and allowable number of cycles for Case 1 and 2 with respect to the base case for the multiobjective optimization corresponding to ω_1_ = 0.5.

Figure 11 shows the Pareto plot for Case 1. Due to a slightly tighter active stress constraint for Case 2, its Pareto plot looks very similar to that of Case 1 and so is not shown here. Instead, Table 6 lists the optimal values of the decision variables for both cases when ω_1_ = 0.5. Interestingly, the trends for tube thickness and tube diameter for Cases 1 and 2 are higher than the base case, as in the results for the single-objective optimization of pressure drop, but the maximum number of tubes in a row is higher than the base case, as in results for the single-objective optimization of metal mass. Corresponding to the optimal design variables presented in Table 6 for Case 1, the pressure drop, metal mass, and allowable number of cycles have reduced by 31.5, 0.52, and 31.9% compared to the base case as shown in Figure 12. Corresponding to the optimal design variables presented in Table 6 for Case 2, the pressure drop has reduced by 37.35% compared to the base case, while the metal mass and allowable number of cycles have increased by 5.25, and 16%, respectively, compared to the base case. For both cases, the optimal results demonstrated that a significant decrease in pressure drops and an increase in the allowable number of cycles can be obtained with a smaller increase in the superheater metal mass. The multioptimization results also show that when the metal mass is decreased, the fatigue damage is increased and the allowable number of cycles is reduced; however, the pressure drop is significantly lower. Transient profiles of the steam outlet temperature, maximum stress, and pressure drop for baseline versus Cases 1 and 2 can be found in the Supporting Information in Figures S1.7–S1.9.

**Table 6:** Optimal Design of the Superheater for the Multiobjective Optimization Corresponding to ω_1_ = 0.5

case	unit	base case	case 1	case 2
tube thickness	mm	3.81	4.06	4.28
tube diameter	mm	36.83	39.02	40.24
tube length	m	7.68	6.85	6.85
maximum number of tubes in a row (surrogate variables for the max width)		88	96	96
number of inlet tube rows		4	4	4

## Conclusions

This study has presented an approach to the optimal design of the primary superheater for power plants increasingly subjected to load-following operations. Two single-objective optimization problems are solved: one for minimizing metal mass and another for minimizing the pressure drop. Because these two objective functions are conflicting, a multiobjective optimization problem is also solved. The same constraints for maximum stress, minimum allowable number of cycles, and main steam temperature are considered for all three optimization problems. It is observed that even with a ramp change in the load as high as 10%, all constraints are consistently satisfied by using the design decision variables as degrees of freedom. Furthermore, a notable observation is that the stress constraint significantly affects the results from the optimization problem. For example, for the single-objective minimization of metal mass, tightening the maximum stress constraint results in a reduction in metal mass and an increase in pressure drop compared to the base case. However, the minimum allowable number of cycles is higher when the metal mass is minimized. In addition, it is also observed that in Case 2, where the stress constraint is active, a slight increase in the metal mass leads to a decrease in the pressure drop and an increase in the minimum allowable number of cycles. For example, when the pressure drop is minimized, the pressure drop decreases by about 45%, but the metal mass increases by about 10%. Similarly for the multiobjective optimization, the pressure drop decreases by about 37% at the expense of a 5% increase in metal mass in another specific scenario. To summarize, this study demonstrates the feasibility and benefits of optimizing the design of superheaters to withstand high ramp rates without compromising stress constraints and/or the allowable number of cycles, providing a generic approach that can be applied to other high-temperature heat exchangers susceptible to thermo-mechanical damage caused by temperature and pressure fluctuations during load-following operations.

The superheater model presented in this work can be found at: https://github.com/IDAES/idaes-pse/blob/main/idaes/models_extra/power_generation/unit_models/boiler_heat_exchanger_2D.py

## Supporting Information Available

Additional results for base case vs Case 2 for the single-objective optimization problems and additional results for base case vs multiobjective optimization problems (PDF)

## Nomenclature

Δ*P*_max_: allowable maximum pressure drops
Δ*P*_total_: total pressure drops
*h*_conv_: convective heat transfer coefficient
*C*_p_: heat capacity
*F*_rad_: gas-surface radiation exchange factor
*Ĥ*: specific enthalpy
*M*_metal_tube_: metal mass
*M*_metal_max_: maximum metal mass
*P*_*x*_: pitch in the parallel direction to the shell-side flow
*P*_*y*_: pitch in the perpendicular direction to the shell-side flow
*T*_m_: mean temperature
*T*_*r*,m_: mean temperature at finite element *r*
*f*_htc,shell_: correction factor for convective heat transfer coefficient due to uneven flow distribution on shell side
*f*_htc,tube_: correction factor for heat transfer coefficient due to uneven distribution of flow inside multiple tubes
*f*_arr_: factor that depends on tube arrangement
*f*_dp,shell_: correction factor due to nonuniform flow distribution on the shell side.
*f*_dp,tube_: correction factor accounting for the roughness of the tubes
*f*_gray_: fraction of gray gas
*k*_*t*_: thermal conductivity of tube
*r*_foul,shell_: fouling resistance on shell side
*r*_foul,tube_: fouling resistance on tube side
ε_gas_: gas emissivity at flue gas temperature
ε_w_: emissivity of fouled tube wall
σ_VonMises_: equivalent von Mises stress
ω_m_: weighting factor for objective “m”
Δ*P*: pressure drops
Δσ: stress range
A: area
E: Young’s modulus
f: fluid
*f*_e_: thickness correction factor
*f*_m_: mean stress correction factor
*f*_s_: surface finish correction factor
*f*_t*_: temperature correction factor
*f*_u_: overall correction factor
*g*: gravitational constant
*h*: heat transfer coefficient
*i*: finite element
*J*: objective function
*k*_e_: elastic correction factor
*K*_f_: effective stress concentration factor
*K*_t_: theoretical stress concentration factor
*k*_v_: plasticity correction factor
*L*: length
*L*_tps_: tube length per segment
*m*: mass
*n*: number of discretization
*N*: number or number of allowable cycles
*N*_t,max_: maximum number of tubes in a row
*N*_row_: number of inlet tube rows
*p*: metric used to measure the distance between the reference point and the objective feasible region
*P*: pressure
*q*: heat flux
*Q*: heat duty
*R*_m_: material tensile strength at room temperature
*t*: time or tube
*T*: temperature
*u*: manipulated variables
w: water
*x*: decision variables
*y*: model output
*z*: length dimension or axial
Δσ_D_: material endurance limit
Δσ_Ri_: reference stress range

## Acronyms

1D: one dimension
2D: two dimension
ALAMO: Automatic Learning of Algebraic Models
ASU: air separation unit
BOFs: basic oxygen furnace
CAPEX: capital expenditure
DAEs: differential algebraic equations
EO: equation-oriented
FEM: finite element method
IAPWS: International association of the properties of water and steam
IDAES: Institute for the Design of Advanced Energy Systems
IPOPT: interior point optimization
MILP: mixed-integer linear programming
MOC: material of construction
MOO: multiobjective optimization
MPC: model predictive control
NGCC: natural gas combined cycle
NLP: nonlinear programming
OPEX: operating expenditure
PDE: partial differential equation
PrSH: primary superheater
SCPC: supercritical pulverized coal-fired
SOO: single-objective optimization

## Greek letters

α: allowable heat duty deviation
β: coefficient of thermal expansion
θ: tangential or parameters
ν: Poisson’s ratio
ρ: density
σ: Stefan–Boltzmann constant or stress
τ: rupture time
ω: weighting parameter
v: velocity
δ: thickness

## Subscripts

conv: convective
fg: flue gas
foul: fouling
fr: friction
grav: gravitational
in: inside
max: maximum
mb: mean beam
min: minimum
nom: nominal
o: outside
*r*: radius or radial
*d*: diameter
rad: radiation
st: steam
wall: tube wall

## References

[ref1] Chen C.; Bollas G. M. Dynamic Optimization of a Subcritical Steam Power Plant under Time-Varying Power Load. Processes 2018, 6, 114 10.3390/pr6080114.

[ref2] Sarda P.; Hedrick E.; Reynolds K.; Bhattacharyya D.; Zitney S. E.; Omell B. Development of a Dynamic Model and Control System for Load-Following Studies of Supercritical Pulverized Coal Power Plants. Processes 2018, 6, 226 10.3390/pr6110226.

[ref3] Wang Y.; Bhattacharyya D.; Turton R. Evaluation of Novel Configurations of Natural Gas Combined Cycle (NGCC) Power Plants for Load-Following Operation Using Dynamic Modeling and Optimization. Energy Fuels 2020, 34 (1), 1053–1070. 10.1021/acs.energyfuels.9b03036.

[ref4] Gonzalez-Salazar M. A.; Kirsten T.; Prchlik L. Review of the Operational Flexibility and Emissions of Gas- and Coal-Fired Power Plants in a Future with Growing Renewables. Renewable Sustainable Energy Rev. 2018, 82, 1497–1513. 10.1016/j.rser.2017.05.278.

[ref5] Wang Y.; Bhattacharyya D.; Turton R. Multiobjective Dynamic Optimization for Optimal Load-Following of Natural Gas Combined Cycle Power Plants under Stress Constraints. Ind. Eng. Chem. Res. 2021, 60 (39), 14251–14270. 10.1021/acs.iecr.1c01461.

[ref6] Fabricius A.; Jackson P. S. Premature Grade 91 Failures - Worldwide Plant Operational Experiences. Eng. Failure Anal. 2016, 66, 398–406. 10.1016/j.engfailanal.2016.04.024.

[ref7] Taler D.; Trojan M.; Dzierwa P.; Kaczmarski K.; Taler J. Numerical Simulation of Convective Superheaters in Steam Boilers. Int. J. Therm. Sci. 2018, 129, 320–333. 10.1016/j.ijthermalsci.2018.03.005.

[ref8] Paterson I. R.; Wilson J. D. Use of Damage Monitoring Systems for Component Life Optimisation in Power Plant. Int. J. Pressure Vessels Piping 2002, 79, 541–547. 10.1016/S0308-0161(02)00094-7.

[ref9] Armor A. F.; Shor S. W. W.; DiDomenico P. N.; Bennett W. E.; Smith L. P. Dynamic Performance of Fossil-Fueled Power Plants. IEEE Trans. Power Appar. Syst. 1982, PAS-101, 4136–4146. 10.1109/TPAS.1982.317092.

[ref10] Trojan M.; Taler D.; Taler J.; Dzierwa P. Modeling of Superheater Operation in a Steam Boiler, ASME 2014 Power Conference; American Society of Mechanical Engineers, 2014.

[ref11] Taler D.; Trojan M.; Taler J. M. Mathematical Modeling of Cross-Flow Tube Heat Exchangers with a Complex Flow Arrangement. Heat Transfer Eng. 2014, 35 (14–15), 1334–1343. 10.1080/01457632.2013.876874.

[ref12] Trojan M.; Taler D. Thermal Simulation of Superheaters Taking into Account the Processes Occurring on the Side of the Steam and Flue Gas. Fuel 2015, 150, 75–87. 10.1016/j.fuel.2015.01.095.

[ref13] Rousseau P. G.; Gwebu E. Z. Modelling of a Superheater Heat Exchanger with Complex Flow Arrangement Including Flow and Temperature Maldistribution. Heat Transfer Eng. 2019, 40 (11), 862–878. 10.1080/01457632.2018.1446816.

[ref14] Granda M.; Trojan M.; Taler D. CFD Analysis of Steam Superheater Operation in Steady and Transient State. Energy 2020, 199, 117423 10.1016/j.energy.2020.117423.

[ref15] Madejski P.; Taler D. Analysis of Temperature and Stress Distribution of Superheater Tubes after Attemperation or Sootblower Activation. Energy Convers. Manag. 2013, 71, 131–137. 10.1016/j.enconman.2013.03.025.

[ref16] Jaremkiewicz M.; Taler D.; Dzierwa P.; Taler J. Determination of Transient Fluid Temperature and Thermal Stresses in Pressure Thick-Walled Elements Using a New Design Thermometer. Energies 2019, 12 (2), 222 10.3390/en12020222.

[ref17] Jaremkiewicz M.; Dzierwa P.; Taler D.; Taler J. Monitoring of Transient Thermal Stresses in Pressure Components of Steam Boilers Using an Innovative Technique for Measuring the Fluid Temperature. Energy 2019, 175, 139–150. 10.1016/j.energy.2019.03.049.

[ref18] Yasniy O.; Pyndus Y.; Iasnii V.; Lapusta Y. Residual Lifetime Assessment of Thermal Power Plant Superheater Header. Eng. Failure Anal. 2017, 82, 390–403. 10.1016/j.engfailanal.2017.07.028.

[ref19] Farragher T. P.; Scully S.; O’Dowd N. P.; Leen S. B. Development of Life Assessment Procedures for Power Plant Headers Operated under Flexible Loading Scenarios. Int. J. Fatigue 2013, 49, 50–61. 10.1016/j.ijfatigue.2012.12.007.

[ref20] Benato A.; Bracco S.; Stoppato A.; Mirandola A. LTE: A Procedure to Predict Power Plants Dynamic Behaviour and Components Lifetime Reduction during Transient Operation. Appl. Energy 2016, 162, 880–891. 10.1016/j.apenergy.2015.10.162.

[ref21] Benato A.; Stoppato A.; Mirandola A. Dynamic Behaviour Analysis of a Three Pressure Level Heat Recovery Steam Generator during Transient Operation. Energy 2015, 90, 1595–1605. 10.1016/j.energy.2015.06.117.

[ref22] Kim R.; Wang Y.; Vudata S. P.; Bhattacharyya D.; Lima F. V.; Turton R. Dynamic Optimal Dispatch of Energy Systems with Intermittent Renewables and Damage Model. Mathematics 2020, 8, 868 10.3390/math8060868.

[ref23] Pattison R. C.; Touretzky C. R.; Johansson T.; Harjunkoski I.; Baldea M. Optimal Process Operations in Fast-Changing Electricity Markets: Framework for Scheduling with Low-Order Dynamic Models and an Air Separation Application. Ind. Eng. Chem. Res. 2016, 55 (16), 4562–4584. 10.1021/acs.iecr.5b03499.

[ref24] Dering D.; Swartz C. L. E.; Dogan N. A Dynamic Optimization Framework for Basic Oxygen Furnace Operation. Chem. Eng. Sci. 2021, 241, 116653 10.1016/j.ces.2021.116653.

[ref25] Yancy-Caballero D. M.; Biegler L. T.; Guirardello R. Large-Scale DAE-Constrained Optimization Applied to a Modified Spouted Bed Reactor for Ethylene Production from Methane. Comput. Chem. Eng. 2018, 113, 162–183. 10.1016/j.compchemeng.2018.03.017.

[ref26] Leipold J.; Seidel C.; Nikolic D.; Seidel-Morgenstern A.; Kienle A. Optimization of Methanol Synthesis under Forced Periodic Operation in Isothermal Fixed-Bed Reactors. Comput. Chem. Eng. 2023, 175, 108285 10.1016/j.compchemeng.2023.108285.

[ref27] Bremer J.; Rätze K. H. G.; Sundmacher K. CO2Methanation: Optimal Start-up Control of a Fixed-Bed Reactor for Power-to-Gas Applications. AIChE J. 2017, 63 (1), 23–31. 10.1002/aic.15496.

[ref28] Alobaid F.; Mertens N.; Starkloff R.; Lanz T.; Heinze C.; Epple B. Progress in Dynamic Simulation of Thermal Power Plants. Prog. Energy Combust. Sci. 2017, 59, 79–162. 10.1016/j.pecs.2016.11.001.

[ref29] Kruger K.; Rode M.; Franke R. In Optimal Control for Fast Boiler Start-up Based on a Nonlinear Model and Considering the Thermal Stress on Thick-Walled Components, IEEE Conference on Control Technology and Applications (CCTA); IEEE, 2002.

[ref30] Kim T. S.; Lee D. K.; Ro S. T. Analysis of Thermal Stress Evolution in the Steam Drum during Start-up of a Heat Recovery Steam Generator. Appl. Therm. Eng. 2000, 20 (11), 977–992. 10.1016/S1359-4311(99)00081-2.

[ref31] Rúa J.; Agromayor R.; Hillestad M.; Nord L. O. Optimal Dynamic Operation of Natural Gas Combined Cycles Accounting for Stresses in Thick-Walled Components. Appl. Therm. Eng. 2020, 170, 114858 10.1016/j.applthermaleng.2019.114858.

[ref32] Rúa J.; Nord L. O. Optimal Control of Flexible Natural Gas Combined Cycles with Stress Monitoring: Linear vs Nonlinear Model Predictive Control. Appl. Energy 2020, 265, 114820 10.1016/j.apenergy.2020.114820.

[ref33] Ma J.; Zamarripa M. A.; Eslick J. C.; Le Q. M.; Bhattacharyya D.; Biegler L. T.; Zitney S. E.; Burgard A. P.; Miller D. C. Dynamic Simulation and Optimization of a Subcritical Coal-Fired Power Plant During Load- Ramping Operations. In Computer Aided Chemical Engineering; Yamashita Y.; Kano M. B. T.-C. A. C. E., Eds.; Elsevier, 2022; Vol. 49, pp 1033–1038.

[ref34] Jiang Y.; Liese E.; Zitney S. E.; Bhattacharyya D. Optimal Design of Microtube Recuperators for an Indirect Supercritical Carbon Dioxide Recompression Closed Brayton Cycle. Appl. Energy 2018, 216, 634–648. 10.1016/j.apenergy.2018.02.082.

[ref35] Jiang Y.; Liese E.; Zitney S. E.; Bhattacharyya D. Design and Dynamic Modeling of Printed Circuit Heat Exchangers for Supercritical Carbon Dioxide Brayton Power Cycles. Appl. Energy 2018, 231, 1019–1032. 10.1016/j.apenergy.2018.09.193.

[ref36] Hedrick K.; Hedrick E.; Omell B.; Zitney S. E.; Bhattacharyya D. Dynamic Modeling, Parameter Estimation, and Data Reconciliation of a Supercritical Pulverized Coal-Fired Boiler. Ind. Eng. Chem. Res. 2022, 61 (45), 16764–16779. 10.1021/acs.iecr.2c01977.

[ref37] Bird R. B.; Stewart W. E.; Lightfoot E. N. Transport Phenomena; Wiley: 2006.

[ref38] Kays W. M.; London A. L. Compact Heat Exchangers; Krieger Publishing Company, 1998.

[ref39] Cozad A.; Sahinidis N. V.; Miller D. C. Learning Surrogate Models for Simulation-Based Optimization. AIChE J. 2014, 60 (6), 2211–2227. 10.1002/aic.14418.

[ref40] Grosshandler W. L. Radcal -- a Narrow-Band Model for Radiation Calculations in a Combustion Environment, NIST 1993.

[ref41] Ma J.; Eason J. P.; Dowling A. W.; Biegler L. T.; Miller D. C. Development of a First-Principles Hybrid Boiler Model for Oxy-Combustion Power Generation System. Int. J. Greenhouse Gas Control 2016, 46, 136–157. 10.1016/j.ijggc.2015.12.036.

[ref42] Modest M. F.; Mazumder S. Radiative Heat Transfer, 3rd ed.; Elsevier Science: New York, NY, 2013.

[ref43] Hottel H. C.; Sarofim A. F. Radiative Transfer; McGraw-Hill Professional: New York, NY, 1967.

[ref44] Holman J. P. Heat Transfer; McGraw-Hill Kogakusha, 1976.

[ref45] Disks, Cylinders, and Spheres BT - Thermal Stresses – Advanced Theory and Applications; Hetnarski R. B.; Eslami M. R., Eds.; Springer Netherlands: Dordrecht, 2009; pp 253–316.

[ref46] Bracco S. Dynamic Simulation of Combined Cycles Operating in Transient Conditions: An Innovative Approach to Determine the Steam Drums Life Consumption, Proceedings of the 25th International Conference on Efficiency, Cost, Optimization and Simulation of Energy Conversion Systems and Processes; ECOS, 2012.

[ref47] Mirandola A.; Stoppato A.; Lo Casto E. Evaluation of the Effects of the Operation Strategy of a Steam Power Plant on the Residual Life of Its Devices. Energy 2010, 35, 1024–1032. 10.1016/j.energy.2009.06.024.

[ref48] Taler J.; Duda P. Solving Direct and Inverse Heat Conduction Problems; Springer: Berlin Heidelberg, 2006.

[ref49] Biegler L. T. Nonlinear Programming: Concepts, Algorithms and Applications to Chemical Processes; SIAM, 2010.

[ref50] Lee A.; Ghouse J. H.; Eslick J. C.; Laird C. D.; Siirola J. D.; Zamarripa M. A.; Gunter D.; Shinn J. H.; Dowling A. W.; Bhattacharyya D.; Biegler L. T.; Burgard A. P.; Miller D. C. The IDAES Process Modeling Framework and Model Library—Flexibility for Process Simulation and Optimization. J. Adv. Manuf. Process. 2021, 3 (3), e10095 10.1002/amp2.10095.

[ref51] Eslick J. C.; Zamarripa M. A.; Ma J.; Wang M.; Bhattacharya I.; Rychener B.; Pinkston P.; Bhattacharyya D.; Zitney S. E.; Burgard A. P.; Miller D. C. Predictive Modeling of a Subcritical Pulverized-Coal Power Plant for Optimization: Parameter Estimation, Validation, and Application. Appl. Energy 2022, 319, 119226 10.1016/j.apenergy.2022.119226.

[ref52] Nicholson B.; Siirola J. D.; Watson J.-P.; Zavala V. M.; Biegler L. T. Pyomo.Dae: A Modeling and Automatic Discretization Framework for Optimization with Differential and Algebraic Equations. Math. Program. Comput. 2018, 10 (2), 187–223. 10.1007/s12532-017-0127-0.

[ref53] Wächter A.; Biegler L. T. On the Implementation of an Interior-Point Filter Line-Search Algorithm for Large-Scale Nonlinear Programming. Math. Program. 2006, 106 (1), 25–57. 10.1007/s10107-004-0559-y.

