# Supplementary material for: Design and Multiobjective Dynamic Optimization of Superheaters for Load-Following Operation in Pulverized Coal Power Plants
Source: Ind Eng Chem Res. 2023 Dec 20;63(1):330–44. doi: 10.1021/acs.iecr.3c02130 (PMC10785806; doi:10.1021/acs.iecr.3c02130)
Supplement: Supplementary file 1 — ie3c02130_si_001.pdf [file ie3c02130_si_001.pdf]

Design and Multi-Objective Dynamic Optimization of Superheaters for Load-Following Operation in Pulverized Coal Power Plants

Quang Minh Le[1], Jinliang Ma[2], Debangsu Bhattacharyya[1*], Stephen E. Zitney[1,3], Anthony P. Burgard[2]

[1] Department of Chemical and Biomedical Engineering, West Virginia University,

Morgantown, WV 26506, USA

[2] National Energy Technology Laboratory, Pittsburgh, PA 15236, USA

[3] National Energy Technology Laboratory, Morgantown, WV 26507, USA

Additional results for base case vs Case 2 for the single objective optimization problems:

Steam outlet temperature profile, maximum stress profiles, and pressure drops profiles for base case vs Case 2 for the single objective optimization of metal mass are shown in Figure S1.1, Figure S1.2, and Figure S1.3, respectively.

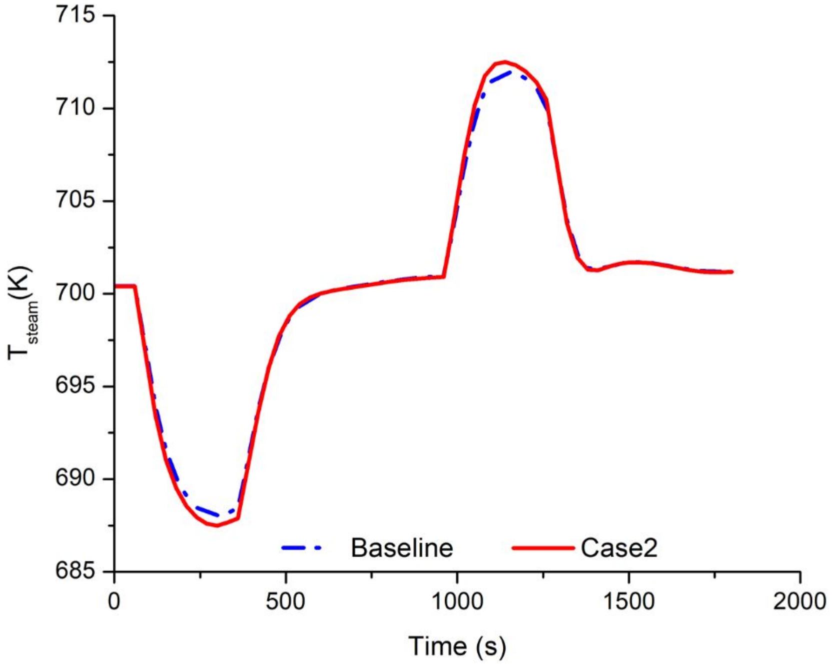

Figure S1.1. Steam outlet temperature profile for base case vs Case 2 for the single objective optimization of the metal mass

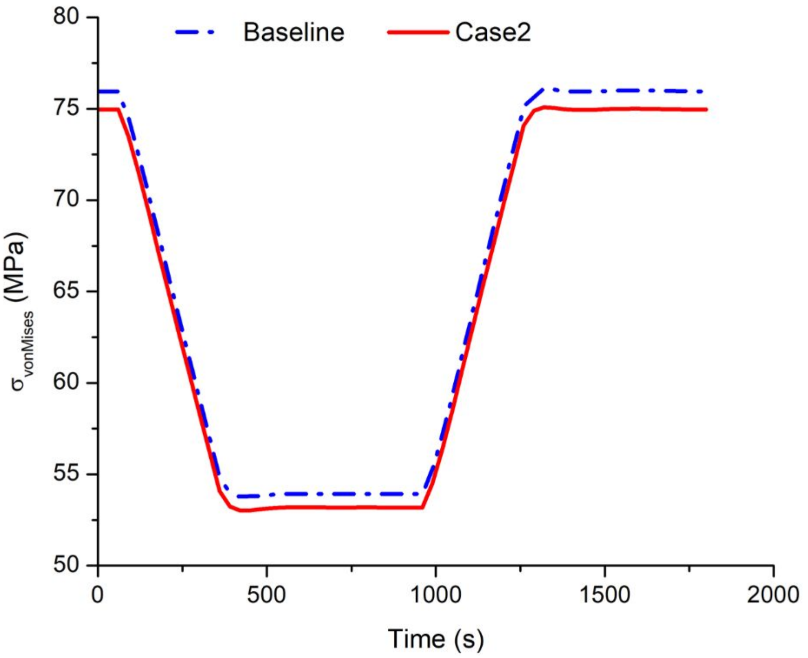

Figure S1.2. Maximum stress profiles at the inlet location for base case vs Case 2 for the single objective optimization of the metal mass

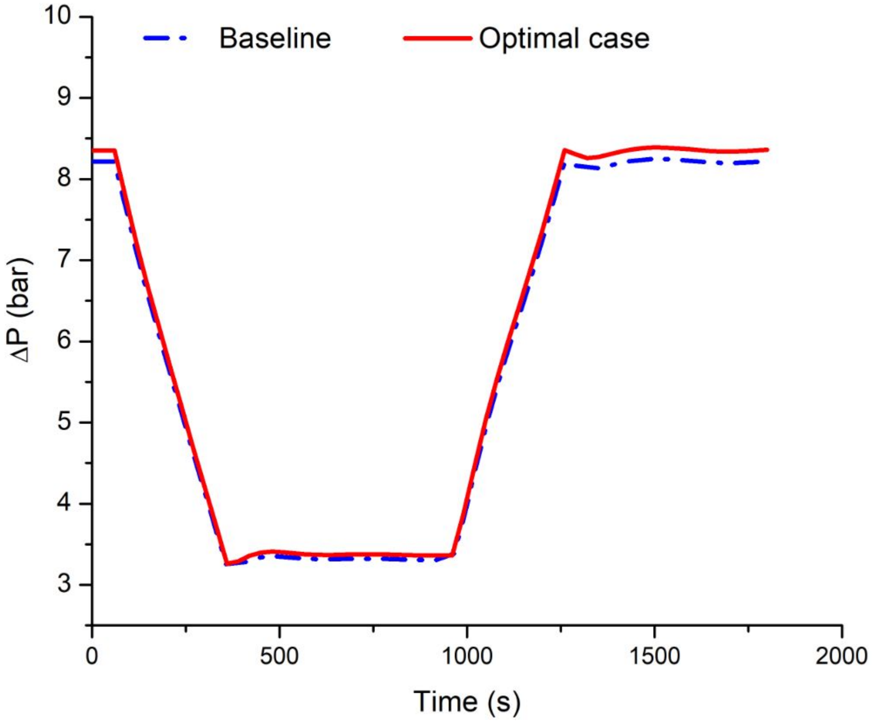

Figure S1.3. Pressure drop profiles for base case vs Case 2 for the single objective optimization of the metal mass

Steam outlet temperature profile, maximum stress profiles, and pressure drops profiles for base case vs Case 2 for the single objective optimization of the pressure drop are shown in Figure S1.4, Figure S1.5, and Figure S1.6, respectively.

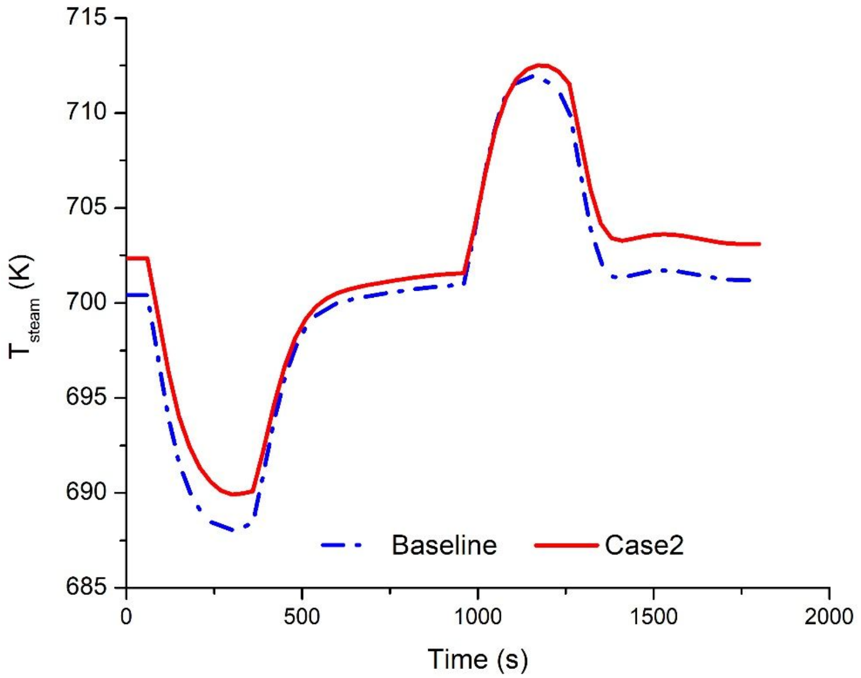

Figure S1.4. Steam outlet temperature profiles for base case vs Case 2 for the single objective optimization of the pressure drop

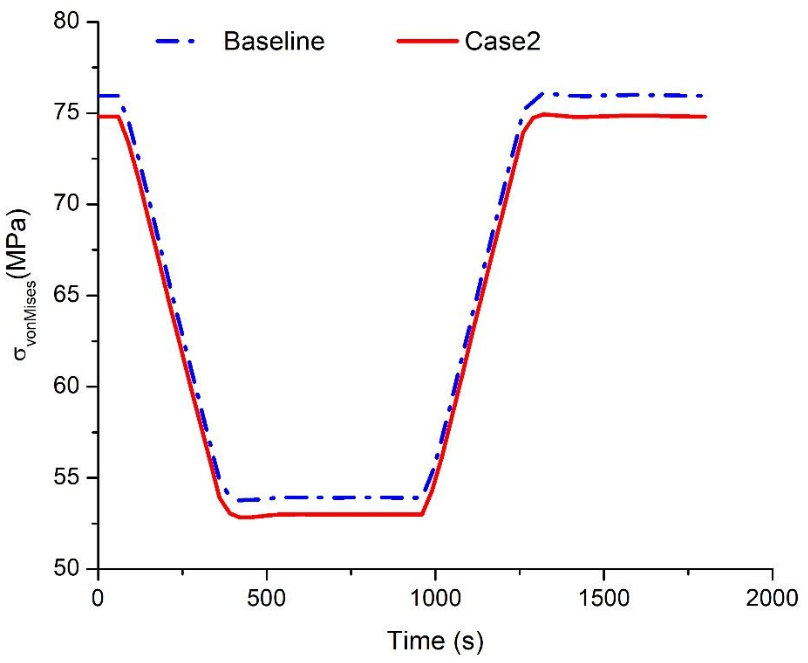

Figure S1.5. Maximum stress profiles for base case vs Case 2 for the single objective optimization of the pressure drop

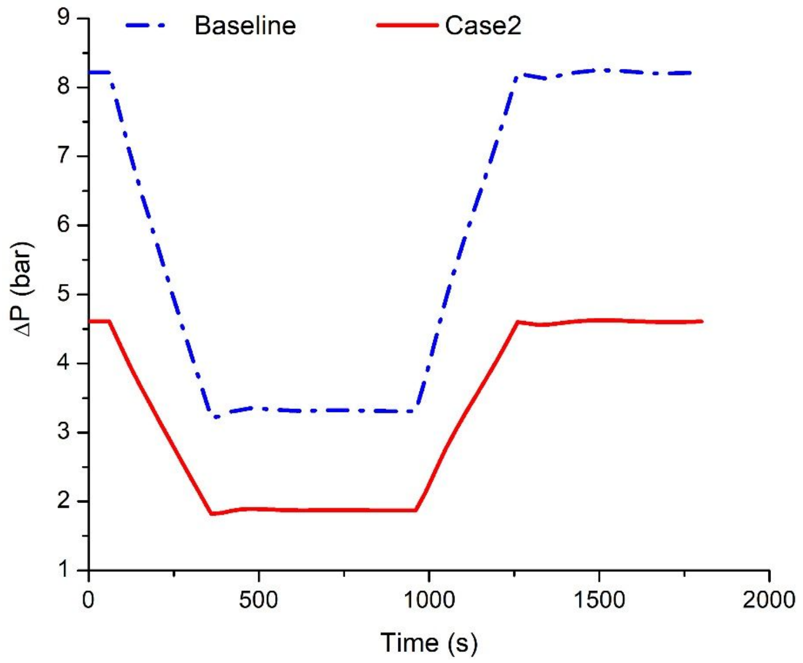

Figure S1.6. Pressure drop profiles for base case vs Case 2 for the single objective optimization of the pressure drop

Additional results for base case vs multi-objective optimization problems:

Steam outlet temperature profile, maximum stress profiles, and pressure drops profiles for base case vs Case 1 and Case 2 for the multi- objective optimization are shown in Figure S1.7, Figure S1.8, and Figure S1.9, respectively.

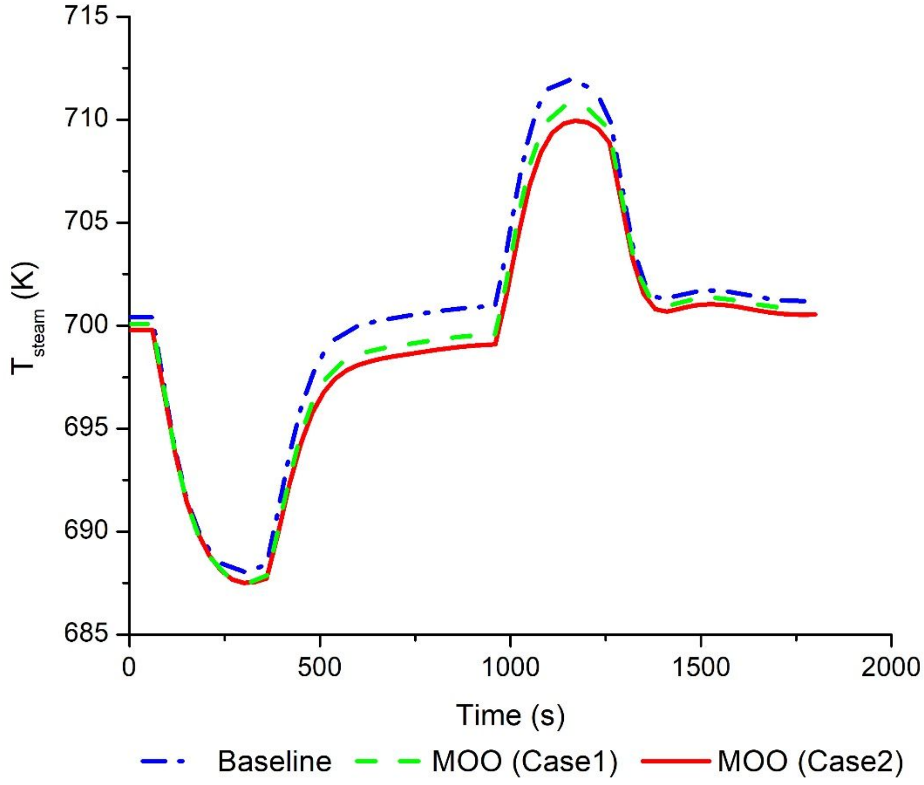

Figure S1.7. Profiles of outlet steam temperature for base case (blue dash-dot line) vs multi-objective optimization Case 1 (green dash line) and Case 2 (red solid line).

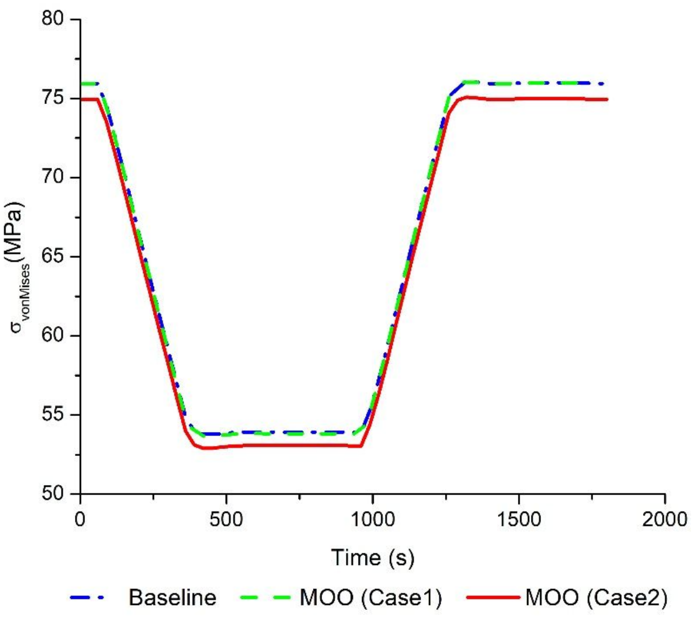

Figure S1.8. Profiles of max stress location for base case (blue dash-dot line) vs multi-objective optimization Case 1 (green dash line) and Case 2 (red solid line).

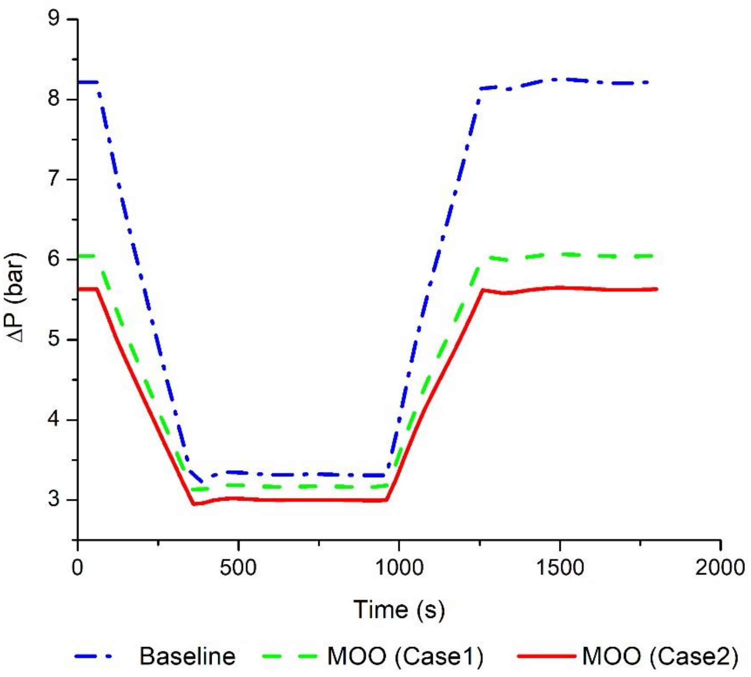

Figure S1.9. Profiles of pressure drop for base case (blue dash-dot line) vs multi-objective optimization Case 1 (green dash line) and Case 2 (red solid line).
